# Intranasal Multiepitope PD‐L1‐siRNA‐Based Nanovaccine: The Next‐Gen COVID‐19 Immunotherapy

**DOI:** 10.1002/advs.202404159

**Published:** 2024-08-08

**Authors:** Rita C. Acúrcio, Ron Kleiner, Daniella Vaskovich‐Koubi, Bárbara Carreira, Yulia Liubomirski, Carolina Palma, Adva Yeheskel, Eilam Yeini, Ana S. Viana, Vera Ferreira, Carlos Araújo, Michael Mor, Natalia T. Freund, Eran Bacharach, João Gonçalves, Mira Toister‐Achituv, Manon Fabregue, Solene Matthieu, Capucine Guerry, Ana Zarubica, Sarit Aviel‐Ronen, Helena F. Florindo, Ronit Satchi‐Fainaro

**Affiliations:** ^1^ Research Institute for Medicines (iMed.ULisboa) Faculty of Pharmacy Universidade de Lisboa Lisbon 1649‐003 Portugal; ^2^ Department of Physiology and Pharmacology Faculty of Medicine Tel Aviv University Tel Aviv 6997801 Israel; ^3^ The Blavatnik Center for Drug Discovery Tel Aviv University Tel Aviv 6997801 Israel; ^4^ Center of Chemistry and Biochemistry Faculty of Sciences University of Lisbon Lisbon 1749‐016 Portugal; ^5^ Department of Clinical Microbiology and Immunology Faculty of Medicine Tel Aviv University Tel Aviv 6997801 Israel; ^6^ The Shmunis School of Biomedicine and Cancer Research George S. Wise Faculty of Life Sciences Tel Aviv University Tel Aviv 6997801 Israel; ^7^ Inter‐Lab, a subsidiary of Merck KGaA, South Industrial Area Yavne 8122004 Israel; ^8^ Centre d'Immunophénomique Aix Marseille Université Inserm, CNRS, PHENOMIN Marseille 13284 France; ^9^ Adelson School of Medicine Ariel University Ariel 4070000 Israel; ^10^ Sagol School of Neuroscience Tel Aviv University Tel Aviv 6997801 Israel

**Keywords:** Dendritic cells, Intranasal, MHC class I and MHC class II peptides, Nanovaccines, PD‐1/PD‐L1 immune checkpoints, SARS‐CoV‐2, siRNA

## Abstract

The first approved vaccines for human use against severe acute respiratory syndrome coronavirus 2 (SARS‐CoV‐2) are nanotechnology‐based. Although they are modular, rapidly produced, and can reduce disease severity, the currently available vaccines are restricted in preventing infection, stressing the global demand for novel preventive vaccine technologies. Bearing this in mind, we set out to develop a flexible nanovaccine platform for nasal administration to induce mucosal immunity, which is fundamental for optimal protection against respiratory virus infection. The next‐generation multiepitope nanovaccines co‐deliver immunogenic peptides, selected by an immunoinformatic workflow, along with adjuvants and regulators of the PD‐L1 expression. As a case study, we focused on SARS‐CoV‐2 peptides as relevant antigens to validate the approach. This platform can evoke both local and systemic cellular‐ and humoral‐specific responses against SARS‐CoV‐2. This led to the secretion of immunoglobulin A (IgA), capable of neutralizing SARS‐CoV‐2, including variants of concern, following a heterologous immunization strategy. Considering the limitations of the required cold chain distribution for current nanotechnology‐based vaccines, it is shown that the lyophilized nanovaccine is stable for long‐term at room temperature and retains its in vivo efficacy upon reconstitution. This makes it particularly relevant for developing countries and offers a modular system adaptable to future viral threats.

## Graphical abstract

Next‐generation thermostable multiepitope nanovaccine generates mucosal immunity and regulates PD‐1/PD‐L1 axis within the dendritic cell (DC)‐T cell interface, coordinating CD4 and CD8 T‐cell immunities and activating robust neutralizing antibody responses against SARS‐CoV‐2.

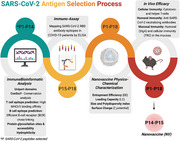

## 1 Introduction

Nanotechnology‐based vaccines were at the forefront of the coronavirus disease of 2019 (COVID‐19) vaccination campaign, with ≈13.59B doses administered globally.^[^ ^1^ ^]^ These vaccines have played a paramount role against severe acute respiratory syndrome coronavirus 2 (SARS‐CoV‐2) infection^[^ ^2^ ^]^ by enabling rapid scale‐up production and manufacturing of a synthetic vaccine while controlling the severity of the disease.^[^ ^3^ ^]^ So far, all nanoplatforms approved as COVID‐19 vaccines by the European Medicine Agency (EMA) and the Food and Drug Administration (FDA) incorporate messenger ribonucleic acid (mRNA) to enable Spike protein production by host cells (e.g., Comirnaty by Pfizer‐BioNTech, and Spikevax by Moderna).^[^ ^4^ ^]^ These nanotechnology‐based vaccines hold several advantages over traditional vaccination approaches.^[^ ^3^ ^]^ However, they pose several challenges, such as high manufacturing, handling, and storage costs.^[^ ^5^ ^]^ The requirement for a vaccine cold chain supply (production, distribution, infrastructure for ultra‐cold storage, administration) imposes significant logistic challenges. Therefore, developing an effective vaccine with a long shelf‐life at room temperature (RT), such as lyophilized vaccines, offers a major logistical advantage over mRNA‐based vaccines that require a cold chain.^[^ ^6^ ^]^ RT‐stable vaccines could enhance the global distribution of vaccines, particularly in low‐income countries, where vaccination rates are generally low.^[^ ^7^, ^8^ ^]^ In addition, robust mucosal immunity, in the form of secretory immunoglobulin A (SIgA), neutralizing antibodies in the bronchoalveolar lavage fluid (BALF), and tissue‐resident memory T and B cells (T_RM_ and B_RM_), can neutralize incoming viral particles at the mucosal surface before infecting epithelial cells and respond immediately in case of secondary infections. Therefore, nasal administration of a COVID‐19 vaccine is being explored as a promising strategy to induce tissue‐specific mucosal immunity and thereby block the transmission of SARS‐CoV‐2.^[^ ^9^ ^]^

The application of a peptide‐based vaccine in a heterologous vaccination modality is an alternative strategy that can offer a relatively simple, reliable, and cost‐effective manufacturing process, leading to a stable and effective product.^[^ ^10^ ^]^ Peptide‐based vaccines are stable in their lyophilized form at RT,^[^ ^11^ ^]^ eliminating the need for cold chain storage and distribution. Moreover, delivering peptides as epitopes by nanoparticles (NP), which traffic to distinct intracellular pathways within antigen‐presenting cells (APC), bypasses the need for protein translation, folding, and processing required by mRNA vaccines. Delivery of antigens entrapped in NP to the conjugation site with major histocompatibility complex (MHC) molecules leads to extensive antigen presentation.^[^ ^12^ ^]^ This enables the induction of broad‐spectrum immunity, which is crucial for overcoming infections caused by different SARS‐CoV‐2 variants.^[^ ^10^ ^]^

Dendritic cells (DC), the most effective APC, manipulate innate and adaptive immunities by activating T cells via T‐cell receptor (TCR) recognition and generating B cell responses either directly via B‐cell receptor (BCR) recognition or through T helper cell mediation.^[^ ^13^ ^]^ In addition, studies have shown the potential of polymeric NP for vaccine delivery through distinct immunization routes, including nasal administration.^[^ ^14^ ^]^ These studies support the exploitation of NP‐based platforms to improve peptide delivery by protecting otherwise poorly immunogenic antigens from harsh conditions at mucosal surfaces. Recent preclinical studies have shown encouraging results using a heterologous immunization strategy, with an intranasal (IN) boost following an intramuscular (IM) prime.^[^ ^15^ ^]^ While IN immunization alone showed suboptimal immunogenicity, an IM‐prime/IN‐boost vaccination strengthened systemic immunity and evoked robust mucosal immunity.^[^ ^16^ ^]^

Here, we describe an effective and biodegradable nanovaccine (NV) that generates a protective anti‐SARS‐CoV‐2 host response. Our NV enables the concomitant delivery of reactive SARS‐CoV‐2 T and B cell peptide epitopes, toll‐like receptor (TLR) 9 and 3 agonists (CpG and Poly(I:C), respectively), and a small interfering RNA (siRNA) targeting programmed cell death ligand 1 (PD‐L1) expression on DC. Several studies have reported that the PD‐1/PD‐L1 axis may regulate host immune responses to SARS‐CoV‐2 infection and COVID‐19 pathogenesis, as infected patients showed upregulation of the PD‐1/PD‐L1 axis and related T‐cell dysfunction,^[^ ^17^ ^]^ potentially leading to immunosuppression. Our studies demonstrate that the top‐ranked COVID‐19 peptide‐ and siRNA‐based NV resulted in a strong induction of antigen‐specific T‐ and B‐cell immunities against SARS‐CoV‐2, including neutralizing antibodies that blocked viral infection of the variants of concern (VOC). Moreover, the heterologous immunization (subcutaneous (SC)‐prime/IN‐boost) improved systemic immunity and evoked tissue‐specific responses, resulting in SIgA, T_RM_, and B_RM_ cells at the respiratory mucosa. These findings support the promising application of our NV platform for mucosal vaccine development.

## 2 Peptide‐Based NV Development

Antigens constitute a major component of a vaccine candidate, dictating the nature of the induced immune response and overall efficacy. An effective protein subunit‐based vaccine includes epitope sequences that can be identified as targets for neutralizing antibodies and for stimulating T‐cell expansion, following their recognition and presentation by APC. T cell‐specific epitopes are short peptide (8‐11 amino acids) and longer peptide (11–30 residues) sequences that are loaded onto MHC class I (MHC‐I) and class II (MHC‐II) proteins, respectively. Upon translocation to the DC surface, the loaded MHC‐I and MHC‐II molecules bind to TCR on CD8^+^ or CD4^+^ T cells, respectively, which are specific for the epitope antigen.^[^ ^18^ ^]^

**Figure 1:**
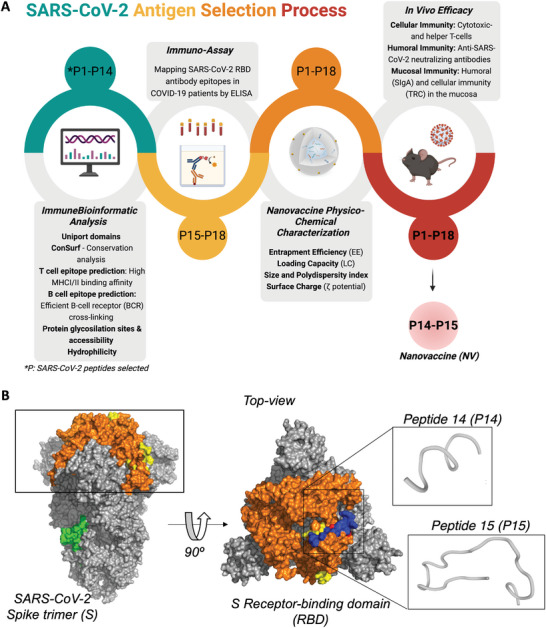
SARS‐CoV‐2 peptide selection immunoinformatic analysis workflow. A) SARS‐CoV‐2 antigen selection strategy. B) SARS‐CoV‐2 Spike trimer (PDB ID 6VXX) surface representation in gray. The receptor‐binding domain (RBD) from each monomer is highlighted in orange. The top‐ranked epitope sequences of in silico workflow are highlighted in yellow (RBD area) and green (other spike regions). In the top view, selected peptides are highlighted in red (MHC‐I binding peptide) and blue (MHC‐II binding peptide). 3D structure prediction of SARS‐CoV‐2 RBD‐peptide (P) 14 and P15 using PEP‐FOLD server.^[^ ^21^ ^]^

In this study, we exploited an immunoinformatic analysis to assist the development of our NV by identifying highly immunogenic MHC‐I and MHC‐II restricted peptide epitopes (**Figure** **1**A). We performed an in silico orthogonal prediction analysis following in‐house criteria: i) sequence accessibility, ii) low frequency of reported mutations, iii) conserved regions estimated to have high population coverage based on their ability to bind with high affinity to multiple human leukocyte antigen (HLA) allotypes, iv) minimum glycosylation sites to avoid masking epitope antigenic determinants by sugars, v) experimental data extracted from the Immune Epitope Database and Analysis Resource (IEDB‐AR) for T cell, B cell, and MHC ligand assays (Figure S1A, Supporting Information).^[^ ^19^ ^]^ Utilizing this pipeline, focusing on SARS‐CoV‐2 as an infectious disease model, enabled us to identify highly immunogenic components with the potential to elicit strong and prolonged cellular and humoral responses. The identified motifs were mainly found in SARS‐CoV‐2 structural proteins, namely the spike (S), nucleocapsid (N), and membrane (M).^[^ ^20^ ^]^

The identified epitopes were subsequently ranked according to hydrophobicity and molecular weight to select those suitable for synthesis and with solubility appropriate for formulating the multi‐epitope‐based nanoparticulate vaccine. Our in silico analysis screened over 100 peptide sequences. The selected 18 top‐ranked SARS‐CoV‐2 potential antigenic epitope motifs (summarized in Table S1, Supporting Information) were mainly localized in the receptor‐binding domain (RBD) of the S protein (Figure 1B).

Based on this observation, we synthesized a linear RBD‐peptide library of 19 long‐peptide sequences (summarized in Table S2, Supporting Information) to map RBD epitope reactivity and specificity to antibodies present in the plasma of patients with severe or critical COVID‐19 illness at the time of blood collection by Enzyme‐Linked Immunosorbent Assay (ELISA). Among the 19 epitopes, one was identified as the most dominant, with the highest reactivity found in 37 out of 42 patient samples (88%) (Figure S1B, Supporting Information). Thus, this peptide (P) (P15 in Table S1, Supporting Information) was selected as the top MHC‐II ligand candidate. In addition, three short peptide sequences (P16‐18) from P15 were identified following our immunoinformatic workflow, based on the recent findings indicating an overlap of the MHC‐I and MHC‐II antigen processing pathways supported by MHC‐II inherent polymorphism.^[^ ^22^ ^]^ Therefore, combining MHC‐I and MHC‐II ligands sharing a common amino acid sequence can represent a useful approach to antigen presentation processes that lead to synergistic antibody‐ and cellular‐mediated immune responses. Following the final step of our antigen selection process, we identified the most effective peptide combination, which induced humoral, cellular, and mucosal immunities: peptides 14 and 15 (P14 and P15).

**Figure 2:**
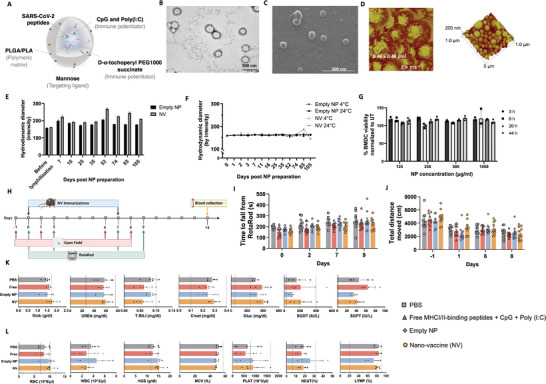
Nanoplatform physicochemical characterization. A) Schematic representation of the nanovaccine (NV). B) Representative image of spherical empty nanoparticle (NP) by transmission electron microscopy (TEM), scale bar = 200 nm. C) Representative image of spherical empty NP by scanning electron microscopy (SEM), scale bar = 1 µm. D) Atomic force microscopy (AFM) images show the spherical shape of NP with a slight roughness surface, scale bar = 200 nm. E,F) Hydrodynamic diameter measurements by dynamic light scattering (DLS) of empty NP (NP w/o immunogens) and NV (NP entrapping one SARS‐CoV‐2‐RBD peptide and the two TLR ligands) over time. E) Lyophilized empty NP and NV stored at 24 °C. F) Empty NP and NV in suspension were stored at 4 and 24 °C. Data represent mean ± s.d. (n =  5). G) Cell viability of bone marrow‐derived dendritic cells (BMDC) after incubation with NP for 44 h by XTT. Data represent mean ± s.d. (a representative graph of 2 independent experiments demonstrating the same trend). All group comparisons were non‐significant (NS) by the One‐way ANOVA test. H) In vivo safety timeline, C57BL/6J mice. I,J) Assessment of motor function. I) RotaRod test. The motor learning of C57BL/6 male mice was analyzed in a five‐lane accelerating RotaRod. J) Open field test. The distance traveled by C57BL/6 male mice during a 15‐min video recording was analyzed using EthoVision 13XT software. Data represent mean ± s.e.m., N = 10 mice. All group comparisons were NS by the One‐way ANOVA test. K,L) Blood test, N = 5 mice. All comparisons between the groups were NS by the One‐way ANOVA test. Data represent mean ± s.e.m. Whisker charts show minimum and maximum values. K) Blood chemistry panel, and L) Blood hematology panel (CBC panel).

For the efficient delivery of the selected SARS‐CoV‐2 antigens, we used our established polymeric‐based nanoplatform (**Figure** **2**A). Previously, we developed a DC‐targeted biodegradable polymeric NP consisting of mannose‐grafted polylactic‐co‐glycolic acid/polylactic acid (PLGA/PLA).^[^ ^23^ ^]^ We^[^ ^23^, ^24^ ^]^ and others^[^ ^25^ ^]^ have demonstrated that incorporating adjuvants within such nanotechnology‐based platforms improves the recognition and delivery of antigens to DC and further regulates the antigen processing pathways to induce stronger antigen‐specific cellular and humoral responses. Based on this knowledge, our established double emulsion solvent evaporation method^[^ ^23^ ^]^ was utilized to develop the NV candidates, where PLGA/PLA NP incorporated the selected SARS‐CoV‐2 peptide sequences, together with CpG and Poly(I:C) oligodeoxynucleotides, which are TLR9 and TLR3 agonists, respectively (Figure 2A). Poly(I:C) was previously shown to synergize with CpG for DC activation by enhancing TLR9 expression on the surface of these APC.^[^ ^26^ ^]^ The NP hydrodynamic diameters ranged between 165 to 274 nm, with a low polydispersity index (Ð), depending on the incorporated peptides (**Table** **1**; Table S3, Supporting Information). Atomic force microscopy (AFM) and electron microscopy demonstrated a uniform spherical morphology with a slightly rough surface (Figure 2B–D). In addition, the NV presented a peptide entrapment efficiency (EE) range of 54.7 ± 1.4% to 99.5 ± 0.1%, depending on the peptide properties. High levels of EE were also quantified for the distinct oligonucleotide‐based immune modulators (CpG and Poly(I:C)) (Table 1; Table S3, Supporting Information).

**Table 1:** Nanoparticle (NP) size, polydispersity index (Ð), entrapment efficiency (EE) of antigens and adjuvants into NP. The EE of peptides (peptides 14 (P14) and 15 (P15)) were determined by the fluorescamine assay (mean ± s.d.; *N* = 3, *n* = 3).

NP	Size^1^ [nm ± s.d.^2^]	Ð ± s.d.^2^	ζ‐Potential [mV ± s.d.^2^]	Peptide EE [% ± s.d.^2^]	Poly(I:C) EE [% ± s.d.^2^]	CpG EE [% ± s.d.^2^]
Empty NP	172 ± 13	0.20 ± 0.02	−8.8 ± 4.3	NA	NA	NA
P14‐loaded NP	203 ± 1	0.15 ± 0.01	−34.2 ± 1.7	74.0 ± 0.5	90.4 ± 2.28	94.4 ± 1.5
P15‐loaded NP	188 ± 8	0.15 ± 0.06	−34.4 ± 1.6	58.0 ± 6.0	93.3 ± 0.75	93.8 ± 1.4

NP – Nanoparticle; Ð – Polydispersity index; EE – Entrapment Efficiency; NA – Not Applicable. ^1^ Z‐average hydrodynamic diameter. ^2^ s.d., standard deviation, obtained from 3 independent batches (*N* = 3).

## 3 Stability and Safety Characteristics of NV

Empty NP and NV formulations were lyophilized using 5% trehalose as a cryoprotectant to assess their suitability for storage as a powder at RT (24 °C). To this end, we evaluated the NP mean diameters, Ð, and zeta (ζ) potential at different time points after lyophilization (Figure 2E; Figure S2A,B, Supporting Information). Additionally, the physicochemical properties of NP and NV stored as suspensions in phosphate‐buffered saline (PBS) were assessed at 4 °C or 24 °C over 3.5 months (Figure 2F). The physicochemical properties of the NP remained close to the target specification (200 nm, Ð < 0.2) over time, whether stored as a powder at 24 °C (Figure 2E; Figure S2A,B, Supporting Information), or a suspension at both 4 °C or 24 °C (Figure 2F; Figure S2C,D).

To evaluate the physiological biocompatibility and in vivo safety of our empty NP or NV, we performed an in vitro viability assay, in vivo behavior assays, blood chemistry, and complete blood count (CBC) analysis. Bone marrow‐derived dendritic cells (BMDC) viability was evaluated by XTT assay following incubation with empty NP at serial concentrations (125, 250, 500, and 1000 µg ml^−1^) over 44 h. The empty NP did not change BMDC viability at any concentration tested over time, supporting their physiological biocompatibility (Figure 2G). The in vivo behavior assays and blood analyses were conducted following the SC administration of two doses of empty NP or NV to naïve mice, one week apart (Figure 2H). Neither the empty NP nor the NV affected mouse body weight (Figure S3A, Supporting Information), motor coordination, balance, learning, or induced neurotoxicity in RotaRod studies (rotating rod at increasing velocity) (Figure 2I), nor did they cause death (Figure S3B, Supporting Information). Moreover, locomotor and anxiety‐like behavior in mice was not affected in an open field test (Figure 2J). Furthermore, our NV did not lead to significant changes in kidney and liver functions, as shown by blood chemistry analysis (Figure 2K), nor did it cause significant changes in blood counts (Figure 2L). Finally, an escalating NV dose demonstrated that higher NV doses were still safe, as no body weight changes or deaths were observed (Figure S3C,D, Supporting Information).

## 4 Immunization with NV Triggered Cellular and Humoral Responses against SARS‐CoV‐2

**Figure 3:**
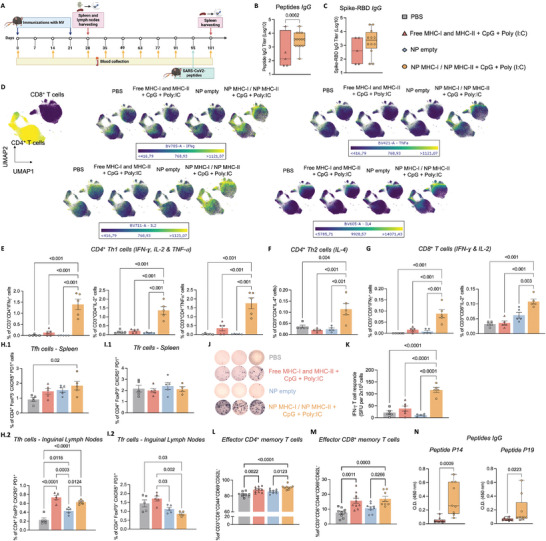
Nanovaccine ‐8 (NV‐8) elicited robust receptor‐binding domain (RDB)‐specific T‐ and B‐cell responses. A) Immunization scheme of C57BL/6J mice timeline. B) SARS‐CoV‐2 RBD‐peptide, and C) RBD‐specific IgG antibody titers determined by Enzyme‐Linked Immunosorbent Assay (ELISA) on day 35. Box and whisker plots represent the mean, min, and max, *N* = 5 (PBS) or 12 (NV), unpaired t‐test. D‐H) Cellular response. D) Uniform manifold approximation and projection (UMAP) plot of lymphocytes (CD45^+^ CD3^+^ cells) colored by Interferon gamma (IFN‐γ), Tumor necrosis factor‐alpha (TNF‐α), Interleukin‐2 (IL‐2), and IL‐4 expression. E) Frequency of antigen‐specific CD4^+^ T cells producing T helper 1 (Th1) cytokines, IFN‐γ, IL‐2, and TNF‐α; F) Th2 cytokine, IL‐4; and G) CD8^+^ T cells producing IFN‐γ and IL‐2 cytokines were evaluated 1 week after the second vaccination (day 28) in 6 h antigen‐stimulated splenocytes. H) Frequency of T_fh_ retrieved from **(1)** spleen and **(2)** inguinal lymph nodes of mice on day 28. Data represent mean ± s.e.m., *N* = 5 mice, one‐way ANOVA followed by Tukey's multiple comparisons test. I) Frequency of T_fr_ cells retrieved from **(1)** spleen and **(2)** inguinal lymph nodes of mice on day 28. Data represent mean ± s.e.m., *N* = 5 mice, one‐way ANOVA followed by Tukey's multiple comparisons test. J) ELISpot representative images of IFN‐γ spot forming cells among splenocytes after *ex vivo* restimulation with peptides 14 and 15 on day 28. Each condition was repeated five times. K) IFN‐γ spot forming units (SFU). Data represent mean ± s.e.m., *N* = 5 mice, one‐way ANOVA followed by Tukey's multiple comparisons test. L‐M) Frequency of effector memory T cells (CD4^+^ and CD8^+^) evaluated 1 week after IV administration of MHCI/II‐binding peptides and the adjuvants (CpG and Poly(I:C)). N) SARS‐CoV‐2 RBD‐peptides IgG levels were evaluated on day 101 by ELISA (dilution 1:500). The number of mice per group: PBS/Empty NP = 9, Free = 10, and NV‐8 = 8, mean ± s.e.m.

Upon characterization of the NP, the eighteen SARS‐CoV‐2 MHC‐I and MHC‐II peptides were incorporated into eleven NV candidates, as described in Table S1 (Supporting Information), together with the TLR agonists CpG and Poly(I:C) for in vivo evaluation. Naïve mice were immunized twice (SC‐Prime/Boost) following the FDA and EMA‐approved schedule for nanotechnology‐based COVID‐19 vaccines^[^ ^27^ ^]^ to characterize the immunostimulatory effects of the NV (**Figure** **3**A).

Cellular immunity was assessed by flow cytometry to measure cytokine production (Interferon gamma, IFN‐γ; Tumor necrosis factor‐alpha, TNF‐α; Interleukin‐2, IL‐2; and Interleukin‐4, IL‐4) in CD8^+^ T cells and T helper (T_h_) cell populations. Moreover, humoral immunity was measured by ELISA to detect circulating high‐affinity antibodies against SARS‐CoV‐2‐RBD and related peptides.

NV‐8, encapsulating P14 and P15 RBD‐derived peptides, induced the most effective SARS‐CoV‐2 humoral and cellular immune responses (Figure S4, Supporting Information). While characterizing the immune profiling on day 28, one week after NV‐booster, there was a significant increase in the secretion of IFN‐γ, TNFα, IL‐2 (Th1‐guided response), and IL‐4 (Th2‐guided response) by CD4^+^ T cells in the spleen of mice immunized with NV‐8 (Figure 3D–F). In addition, NV‐8 induced strong CD8^+^ T cell responses with high expression of intracellular INF‐γ and IL‐2 (Figure 3D,G), as well as T follicular helper (T_fh_) cells (Figure 3H), demonstrating the formulation's potential to mount robust adaptive immunity. On day 35, two weeks after the booster dose, NV‐8‐immunized mice presented the highest IgG titers against SARS‐CoV‐2‐RBD (Figure 3B,C), for antibody kinetics, see Figure S5A, Supporting Information). CD4^+^ T cells, T_fh_, and T follicular regulatory (T_fr_) cells play important roles in germinal center (GC) formation, B‐cell function regulation, and the production of high‐affinity antibodies to a specific antigen. This effect was shown to be mediated by T‐cell cytokine secretion, such as IL‐4.^[^ ^28^ ^]^ Our data indicates a correlation between the increased T_fh_ population, elevated intracellular IL‐4 in CD4^+^ T cells, and the high levels of specific antibody secretion following NV‐8 vaccination (Figure 3H,I).

Importantly, we confirmed the RBD peptide antigen‐specific T‐cell responses using an enzyme‐linked immuno‐spot (ELISpot) assay. The highest overall IFN‐γ production was obtained from splenocytes of NV‐8 vaccinated mice upon stimulation with MHC‐I and MHC‐II‐restricted RBD peptides (Figure 3J,K).

Seventy‐four days after the booster dose, mice were challenged with an intravenous (IV) injection of RBD peptides and adjuvants (Figure 3A), mimicking circulating viral fractions in the blood following SARS‐CoV‐2 infection, to stimulate a similar immune response via TLR.^[^ ^29^ ^]^ Mice immunized with NV‐8 showed expansion in the effector memory T cell (CD4^+^ and CD8^+^) population in the spleen (Figure 3I,M) and significantly higher specific SARS‐CoV‐2 antibody levels 6 days following the challenge intervention (Figure 3N). These results can be correlated to the observed higher IL‐2 levels (Figure 3D,E,G), as elevation in polarizing cytokine levels such as IL‐2 can stimulate naïve CD4^+^ T‐cell proliferation and differentiation into Th1 and Th2 lymphocytes while enhancing T‐cell memory.^[^ ^30^ ^]^

In addition, we found that administering the same antigens and adjuvants in solution (Free) did not induce significant effector CD4^+^ and CD8^+^ T‐cell responses. Both CD4^+^ Th1‐ and CD8^+^ T‐cell populations failed to differentiate into IFN‐γ, TNF‐α, and IL‐2 producers (Figure 3D,E,G). Therefore, successful B‐cell and T‐cell priming and expansion required co‐delivery of RBD peptides and TLR ligands, enabled by our NV. Our platform facilitated multi‐targeting TLR effects on antigen presentation and subsequent antigen‐specific T‐cell activation through CpG and Poly(I:C) delivery to DC. This data aligns with our previous study demonstrating the importance of co‐delivering selected antigens and adjuvants within our nanoplatform for effective DC‐mediated antigen presentation.^[^ ^23^ ^]^ Furthermore, empty NP did not elicit cellular responses (Figure 3D–I) nor induce antibodies against RBD and RBD‐peptide antigens (Figure S5B, Supporting Information).

## 5 Co‐Delivery of SARS‐CoV‐2 Peptide Antigens, TLR Ligands and siRNA Targeting PD‐L1 (siPD‐L1) Potentiates Antibody Immunity

Incorporating cell function modulators such as siRNA in DC vaccines to enhance DC maturation and antigen presentation has been a researched strategy in the past years. Our results, so far, have demonstrated that NV‐8 elicits robust antibody secretion and T‐cell‐mediated immunity. However, we aimed to explore additional immune‐reactive effects by incorporating siRNA in our NV to further test the boundaries of our nanoplatform. Therefore, we chose to downregulate the PD‐1/PD‐L1 axis, as PD‐L1‐silenced DC have been reported to exhibit stimulatory characteristics,^[^ ^31^ ^]^ potentially promoting T‐cell proliferation and subsequent effector functions of B and T lymphocytes.^[^ ^32^ ^]^ PD‐L1 expression on the DC surface is known to increase upon antigen uptake and presentation.^[^ ^33^ ^]^ In addition, it has been implicated in generating T_fr_ cells, which inhibit GC response.^[^ ^34^ ^]^

**Figure 4:**
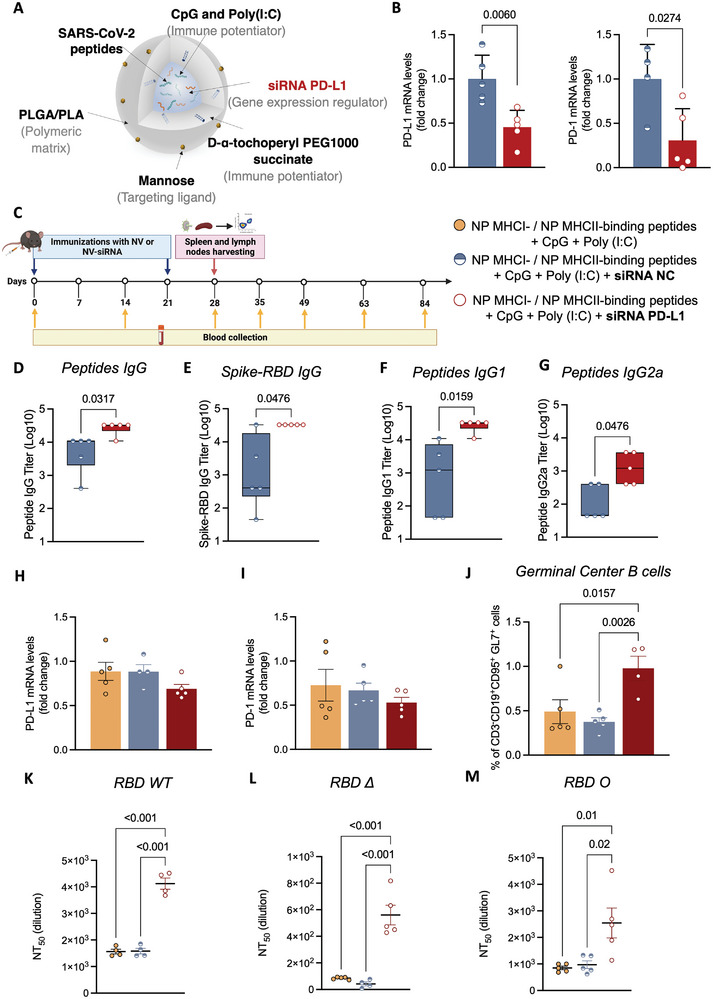
Co‐delivery of receptor‐binding domain (RBD)‐peptides, toll‐like receptor (TLR) ligands, and siRNA against Programmed death‐ligand 1 (PD‐L1) (siPD‐L1 NV‐8) increases NV‐8 neutralizing‐antibody responses. A) Schematic representation of siPD‐L1 NV‐8 B) *PD‐L1* and *PD‐1* mRNA levels from spleen, 55 h post siNC NV‐8 (negative control of scramble siRNA) or siPD‐L1 NV‐8 immunization. mRNA levels measured by qRT‐PCR. Data represent mean ± s.e.m., *N* = 5 mice, unpaired t‐test. C) Immunization scheme of C57BL/6J mice timeline. SARS‐CoV‐2 RBD‐peptides, and RBD‐specific IgG D,E), IgG1 F), and IgG2a G) antibody titers determined by ELISA. Box and whiskers represent the mean, min, and max, *N* = 5 mice per group, unpaired *t*‐test. H,I) *PD‐L1*, and *PD‐1* mRNA levels, from spleens, on day 28. mRNA levels measured by qRT‐PCR. Data represent mean ± s.e.m., *N* = 5 mice, one‐way ANOVA followed by Dunnett's multiple comparisons test. J) Frequency of germinal center (GC) B cells detected by flow cytometry on day 28. Data represent mean ± s.e.m., *N* = 5 mice, one‐way ANOVA followed by Tuke's multiple comparisons test. NT_50_ in serum against SARS‐CoV‐2 RBD WT K), Delta (Δ) L), and Omicron BA.1.1 (Ο) M) in mice immunized with NV‐8, siNC NV‐8, and siPD‐L1 NV‐8 using surrogate virus neutralization test. Data represent mean ± s.e.m., *N* = 5 mice, one‐way ANOVA followed by Dunnett's multiple comparisons test.

Therefore, we hypothesized that downregulating PD‐L1 in DC would improve DC‐T‐B cell interactions during antigen presentation following our NV treatment. To that end, we transiently co‐entrapped a siRNA oligonucleotide targeting PD‐L1 expression in NV‐8 to suppress PD‐1/PD‐L1 signaling at the DC‐T‐B cell interface (**Figure** **4**A; **Table** **2**). Similar to NV‐8, the siPD‐L1 NV‐8 remains stable as a suspension or lyophilized powder (Figure S6, Supporting Information).

**Table 2:** NP size, polydispersity index (Ð), entrapment efficiency (EE), and loading capacity (LC) of antigens into NP. The EE and LC of peptides (peptide 14 (P14) to peptide 15 (P15)) were determined by the fluorescamine assay (mean ± s.d.; N = 3, n = 3). The EE and LC of CpG were determined using the Quant‐iT™ OliGreen™ ssDNA assay kit (mean ± s.d.; N = 3, n = 3). The EE and LC of Poly(I:C) and siRNA were determined using the Quant‐iT™ RNA Assay Kit (broad range) (mean ± s.d.; N = 3, n = 3).

NP	Size^1^ [nm ± s.d.^2^]	Ð ± SD^2^	ζ‐Potential [mV ± s.d.^2^]	Peptide EE [% ± s.d.^2^]	Peptide LC [µg mg^−1^ ± s.d.^2^]	CpG EE [% ± s.d.^2^]	CpG LC [µg mg^−1^ ± s.d.^2^]	Poly(I:C) + siRNA EE [% ± s.d.^2^]	Poly(I:C) + siRNA LC [µg mg^−1^ ± s.d.^2^]
**P14**‐siPD‐L1 loaded NP	204 ± 6	0.15 ± 0.01	−32.4 ± 3.1	74.0 ± 0.5	74.0 ± 0.5	94.6 ± 1.8	3.3 ± 0.08	99.8 ± 0.02	11.4 ± 0.003
**P15**‐siPD‐L1 loaded NP	202 ± 9	0.12 ± 0.01	−32.2 ± 2.1	58 ± 6	58 ± 6	95.2 ± 9.0	3.3 ± 0.2	100 ± 0.04	10.4 ± 0.004

NP – Nanoparticle; Ð – Polydispersity index; EE – Entrapment Efficiency; LC – Loading Capacity.

^1^ Z‐average hydrodynamic diameter. ^2^ SD, standard deviation, obtained from 3 independent batches (*N* = 3).

The ability of siPD‐L1‐loaded NV‐8 to downregulate the expression of this immune checkpoint was validated in vivo by a significant decrease in *PD‐L1* and *PD‐1* mRNA levels, reduced by 50%, quantified in splenocytes of mice immunized with siPD‐L1 NV‐8 compared to mice treated with negative control scrambled siRNA (siNC)‐loaded NV‐8‐treated (Figure 4B). Peptides and oligonucleotides maintained their activity after NV lyophilization (Figure S7, Supporting Information). We further investigated the immunogenicity enhanced by siPD‐L1 incorporation by comparing it with NV‐8 alone in vivo, following the same immunization schedule (Figure 4C). Mice immunized with siPD‐L1 NV‐8 showed increased IgG titers against SARS‐CoV‐2 RBD and the encapsulated RBD peptides (Figure 4D,E). The predominant IgG1 and IgG2a subclass levels against SARS‐CoV‐2 epitopes indicated enhanced immunogenicity in siPD‐L1 NV‐8‐immunized mice (Figure 4F,G). This data is essential as IgG1 is crucial as a Th2‐associated isotype antibody, while IgG2a exhibits stronger Fcγ Receptor (FcγR)‐mediated activity, facilitating viral clearance via antibody‐dependent cellular cytotoxicity.^[^ ^35^ ^]^ Moreover, these mice also presented significant GC B cell proliferation (Figure 4J), essential for antibody diversification and affinity maturation, supporting robust antibody production. Interestingly, a significant reduction in *PD‐1* and *PD‐L1* mRNA levels persisted in splenocytes of siPD‐L1 NV‐8‐immunized mice one week after the booster dose, indicating a sustained systemic response on day 28 (Figure 4H,I).

Consistent with high IgG levels, animals immunized with siPD‐L1 NV‐8 revealed the highest neutralizing antibody (nAb) titers against both SARS‐CoV‐2 WT and VOC (Figure 4K–M). We assessed nAbs using ELISA and a surrogate virus neutralization test (sVNT) with purified RBD of WT or SARS‐CoV‐2 VOC (alpha, beta, delta, gamma, and omicron) and ACE2 as the host cell receptor. Overall, NV formulations (NV‐8, siNC NV‐8, and siPD‐L1 NV‐8) effectively blocked RBD–ACE2 interactions. Importantly, siPD‐L1 NV‐8 exhibited the highest neutralizing antibody titers (NT_50_) against RBD WT and VOC, including omicron (Figure 4K–M; Figure S8, Supporting Information). To the best of our knowledge, this study is the first to report the co‐delivery of SARS‐CoV‐2 peptide antigens, TLR ligands, and siPD‐L1, highlighting their synergistic role in eliciting robust protective antibody‐ and T cell‐mediated immunities against SARS‐CoV‐2. Given the shared mechanisms with other diseases, replacing SARS‐CoV‐2 antigens with antigens from other pathogens could render our NV platform relevant to various pathologies.

## 6 siPD‐L1 NV‐8 Intranasal Booster Elicited Strong Immunogenicity at the Respiratory Mucosa, Inducing Systemic Immune Responses

Immunization routes significantly impact vaccine efficacy.^[^ ^36^ ^]^ Current parenteral COVID‐19 vaccines induce robust cellular responses, leading to high efficacy against severe disease and asymptomatic infection. However, systemic immunity decreases ≈4 months post‐booster dose and fails to confer protection at the infection site.^[^ ^37^ ^]^ Therefore, IN administration of a COVID‐19 vaccine emerges as a promising strategy to block SARS‐CoV‐2 transmission and prevent severe symptoms. Previous studies have shown that a heterologous immunization regimen (IM‐Prime/IN‐Boost) induces a systemic immune response similar to IM‐Prime/Boost, while additionally activating mucosal immunity.^[^ ^16^, ^38^ ^]^ Moreover, T_RM_ from COVID‐19 patients persists for up to 10 months post‐recovery, suggesting their role in providing lasting protective immunity.^[^ ^39^ ^]^

**Figure 5:**
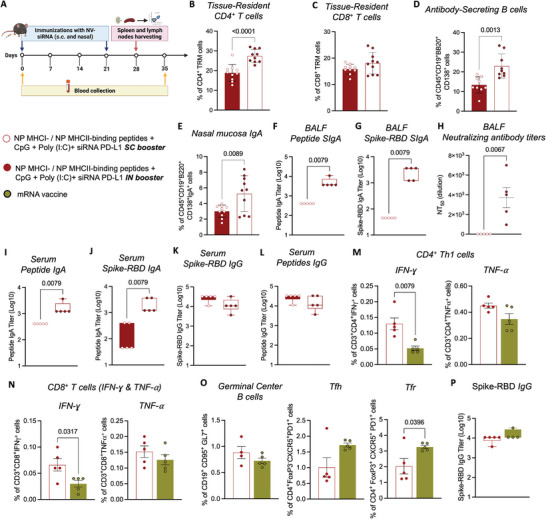
Intranasal siPD‐L1 NV‐8‐booster elicited robust mucosal immunity. A) Immunization scheme of C57BL/6J mice timeline. B,C) Mucosal‐cellular response determined by flow cytometry on day 28. Frequency of tissue‐resident T cells (T_RM_) (CD4^+^ and CD8^+^) D,C), and antibody‐secreting B cells E) in the nasal mucosa. Data represent mean ± s.e.m., *N* = 5 mice (subcutaneous (SC) booster), and 10 (intranasal (IN) booster), one‐way ANOVA followed by Tuke's multiple comparisons test. E–K) Mucosal‐humoral response. E) Frequency of immunoglobulin (Ig) IgA‐B cells in nasal mucosa on day 28. F,G) SARS‐CoV‐2 receptor‐binding domain (RBD)‐peptides and RBD‐specific secretory immunoglobulin A (SIgA) titers in bronchoalveolar lavage fluid (BALF) determined by ELISA. Box and whiskers represent the mean, min, and max, *N* = 5 mice per group, unpaired t‐test. H) Neutralizing antibody titers (NT_50_) against SARS‐CoV‐2 in BALF determined by surrogate virus neutralization test. Data represent mean ± s.e.m., *N* = 5 mice, unpaired t‐test. SARS‐CoV‐2 RBD‐peptides and RBD‐specific IgA titers I,J), and IgG in serum K,L) determined by ELISA. Box and whiskers represent the mean, min, and max, *N* = 5 mice per group, unpaired *t*‐test. M–P) Comparison between siPD‐L1 NV‐8 versus mRNA vaccine. M,N) Cellular response. The frequency of antigen‐specific CD4^+^ T cells producing T helper 1 (Th1) cytokines such as interferon‐gamma (IFN‐γ) and tumor necrosis factor‐alpha (TNF‐α) M) was evaluated one week after the intranasal booster (day 28) and after stimulation with RBD‐peptides or spike protein for 6 h. Cytokine production (IFN‐γ and TNF‐α) was also evaluated on CD8^+^ T cells N). O) Frequency of germinal center B cells, T_fh_, and T_fr_ cells retrieved from mice on day 28. Data represent mean ± s.e.m., *N* = 5 mice, unpaired *t*‐test. P) SARS‐CoV‐2 RBD IgG antibody titers determined on day 35 by ELISA. Box and whisker plots represent the mean, min, and max, *N* = 5, unpaired t‐test.

First, to assess the in vivo safety of siPD‐L1 NV‐8, we conducted behavioral assays and blood analyses following the IN administration of two doses of siPD‐L1 NV‐8 to naïve mice, one week apart (Figure S9A–G, Supporting Information). siPD‐L1 NV‐8 did not affect mouse body weight change, survival, motor coordination and imbalance, locomotor activity, or anxiety‐like behavior compared to PBS‐treated mice (Figure S9A–E, Supporting Information). In addition, there were no significant changes in kidney and liver functions or blood count following IN administration of siPD‐L1 NV‐8 (Figure S9F,G, Supporting Information). Importantly, the IN administration of siPD‐L1 NV‐8 did not cause any damage to the typical respiratory epithelium overlying mucosa morphology, which contains a normal amount of inflammatory cells, without evidence of edema or fibrosis (Figure S9H, Supporting Information). These findings suggest that the IN administration of siPD‐L1 NV‐8 is safe and does not induce adverse effects. Next, we assessed the potential of our siPD‐L1 NV‐8 to trigger mucosal immunity. Mice were immunized with homologous (SC‐Prime/Boost) or heterologous (SC‐Prime/IN‐Boost) regimens (**Figure** **5**A). One week after the booster dose, mucosal cell‐mediated immunity was evaluated using flow cytometry. The immune‐profiling analysis of the nasal cavity revealed a robust mucosal cellular immunity in IN‐boosted‐treated mice, with significantly increased frequencies of CD4^+^ and CD8^+^ T_RM_ cells compared to SC‐boosted‐treated mice (Figure 5B,C). In addition, mice receiving the IN booster displayed the highest numbers of antibody‐secreting B cells and IgA‐secreting B cells, indicative of robust mucosal humoral immunity (Figure 5D,E). On day 35, mucosal humoral immunity was further evaluated by measuring the SIgA levels. Mucosal IgA is crucial for protecting mucosal surfaces by neutralizing respiratory viruses or impeding their attachment to epithelial cells.^[^ ^40^ ^]^ The demonstrated ability of our NV to induce anti‐SARS‐CoV‐2 immunity via IN administration suggests that our nanoplatform could effectively combat other infectious diseases with similar transmission mechanisms by selecting disease‐relevant immunogenic antigens according to our antigen selection guidelines.

Aligned with the activation of cellular immunity, higher levels of SIgA and IgA specific to SARS‐CoV‐2 RBD and RBD peptides were detected in BALF and serum, respectively, in mice receiving the IN‐boost (Figure 5F–J). Importantly, IgG titers were comparable across all immunized animals (Figure 5K,L). Finally, we further analyzed the cellular and humoral responses induced by our siPD‐L1 NV‐8 (SC‐Prime/IN‐Boost) compared to a commercially available mRNA COVID‐19 vaccine (SC‐Prime/Boost). siPD‐L1 NV‐8 elicited a robust Th1‐guided response characterized by TNF‐α and IFN‐γ secretion (Figure 5M), along with a significant increase in IFN‐γ‐producing CD8^+^ T cells (Figure 5N). Additionally, mice treated with siPD‐L1 NV‐8 (IN booster) exhibited enhanced GC responses (Figure 5O), consistent with the strong humoral and mucosal immunities also detected in this group. In contrast, the mRNA vaccine induced higher numbers of T_fh_ and T_fr_ cells (Figure 5O). Regarding antibody levels, both vaccines revealed similar IgG titers (Figure 5P). Overall, our data show that heterologous immunization with siPD‐L1 NV‐8 effectively stimulates mucosal (cellular and humoral) immunity, providing robust protection against SARS‐CoV‐2 infection.

**Figure 6:**
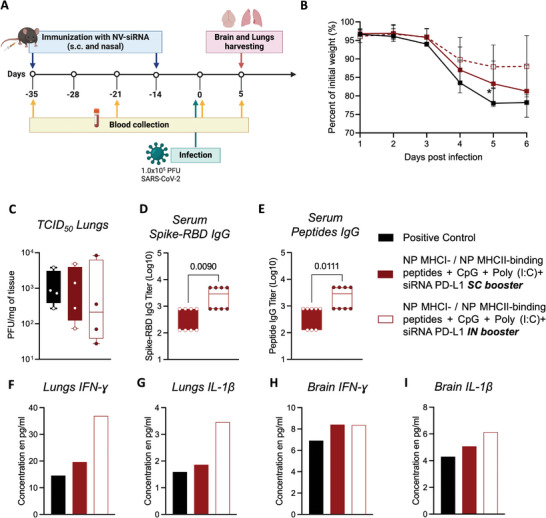
Intranasal siPD‐L1 NV‐8 booster triggers a protective Th1‐guided immune response in vivo. A) Immunization scheme of K18‐hACE2 mice timeline. K18‐hACE2 mice (*N* = 10 or 11) received a SC prime immunization with siPD‐L1 NV‐8. Mice received the SC or IN booster three weeks later. Two weeks after the boost immunization, mice were infected intranasally with 1.0 × 10^5^ PFU SARS‐CoV‐2. B) Animals were monitored for body weight. On day 5 post‐infection, viral loads in the lung tissue C) were determined by an assay to quantify PFUs of infectious SARS‐CoV‐2. D,E) The SARS‐CoV‐2 RBD‐ and RBD peptides‐specific IgG titers in mouse serum were determined by ELISA. Box and whiskers represent the mean, min, and max, *N* = 4 mice per group in duplicate, unpaired *t*‐test. Mouse cytokine array shows high levels of IFN‐γ and IL‐1β in the lungs F,G) and brain H,I).

We further investigated the efficacy of these immune responses in a preclinical lethal model of SARS‐CoV‐2 infection in mice^[^ ^41^ ^]^ following the immunization and challenge schedule shown in **Figure** **6**A. Animals’ weight loss was compatible with clinical scoring of SARS‐CoV‐2 infection. Assessment of weight loss and clinical scoring on day 5 post‐infection indicated reduced initial weight loss in immunized animals, suggesting delayed disease progression (Figure 6B). In addition, viral titers in lung lysates showed a decreasing trend in vaccinated groups (Figure 6C). In transgenic K18‐hACE2 mice, IN administration of SARS‐CoV‐2 results in high viral titers in the brain. This supports the neuroinvasive potential of SARS‐CoV‐2 in our mouse models, which, at high lethal viral doses, exacerbate disease severity and increase mortality. Notably, robust humoral immunity was triggered after two siPD‐L1 NV‐8 doses (IN booster), which is shown by the significantly higher levels of Spike‐RBD‐ (Figure 6D) and RBD peptide‐specific IgG (Figure 6E) detected in the serum. Additionally, higher levels of IFN‐ɣ (Figure 6F,H) and IL‐1β (Figure 6G,I) were quantified in lung and brain lysates of mice vaccinated with siPD‐L1 NV‐8 (IN booster). The increase in these pro‐inflammatory cytokines indicates a Th1‐shifted immune response, crucial for protection against severe SARS‐CoV‐2 infection. This profile is of utmost importance as it was shown that the cellular immunity associated with protection against severe SARS‐CoV‐2 infection requires a robust IFN‐ɣ secretion.^[^ ^42^ ^]^ IFN‐γ is essential for macrophage activation and antigen presentation, promoting T‐cell activation and proliferation, while aiding the clearance of infected cells.^[^ ^42^ ^]^ IL‐1β, on the other hand, is a pro‐inflammatory cytokine that has a role in the initiation and regulation of the immune response. It contributes to immune cell recruitment to the site of infection and adaptive immune response activation.^[^ ^43^ ^]^ Neuroinflammation is associated with cytokine production, which influences neuronal and glial functions. Although proinflammatory cytokines such as IFN‐γ and IL‐1β are thought to be the major mediators of neuroinflammation, their role in brain injury and infection remains disease‐defined. It has been shown that IFN‐γ and IL‐1β induce astrogliosis and microgliosis, enhance the secretion of brain‐derived neurotrophic factor (BDNF), one of the many neurotrophic factors after brain injury or infection, and promote the survival of cortical neurons.^[^ ^44^ ^]^ Taken together, these vaccine‐induced cytokines create a robust immune environment that improves protection against SARS‐CoV‐2 by controlling viral replication and spread within the host.

## 7 Discussion

This study describes the development of a specific, safe, and immunogenic next‐generation multiepitope‐ and siRNA‐based NV against SARS‐CoV‐2. Central to our approach is the critical role of epitope selection in designing a robust protein subunit‐based vaccine for SARS‐CoV‐2. Through immunoinformatic analysis, we identified highly immunogenic MHC‐I and MHC‐II restricted peptide epitopes, prioritizing criteria such as sequence accessibility, low mutation frequency, conservation, minimal glycosylation sites, and corroborated experimental data. This rigorous selection process enabled us to pinpoint epitopes primarily located within the structural proteins of SARS‐CoV‐2, notably the S, N, and M proteins. A significant finding was the identification of 18 top‐ranked SARS‐CoV‐2 epitopes, mainly in the RBD of the S protein. Notably, one epitope, P15, exhibited dominant reactivity in ELISA assays using plasma from severe COVID‐19 patients, highlighting its potential as a prime MHC‐II ligand candidate.

For the delivery of these antigens, we employed a polymeric‐based NP platform, incorporating TLR9 and TLR3 agonists (CpG and Poly(I:C) oligodeoxynucleotides, respectively) to enhance DC activation. Our NP formulations demonstrated optimal physicochemical properties, high peptide entrapment efficiency, stability under various storage conditions, and biocompatibility in vitro and in vivo. This study showcases the modularity of our nanoplatform, which can be readily adapted to elicit immune responses against potentially threatening SARS‐CoV‐2 VOC, by replacing the incorporated SARS‐CoV‐2‐related peptides. Together with the NP's ability to co‐entrap different molecules, our well‐established and robust immunoinformatic workflow facilitates the replacement of current epitope peptide sequences with other immunodominant epitopes from novel SARS‐CoV‐2 variants and other infectious diseases. Immunization with our NV candidates, particularly NV‐8 encapsulating P14 and P15, elicited potent cellular and humoral responses against SARS‐CoV‐2 variants, including VOC (delta and omicron). Mice vaccinated with NV‐8 showed increased cytokine secretion (IFN‐γ, TNF‐α, IL‐2, IL‐4) and high IgG titers specific to SARS‐CoV‐2 RBD, indicating robust Th1 and Th2 responses and effective T‐cell memory. Notably, the co‐delivery of SARS‐CoV‐2 antigens, TLR ligands, and siRNA targeting PD‐L1 further potentiated antibody responses, suggesting that PD‐L1 downregulation enhances DC‐T‐B cell interactions and promotes GC reactions.

The versatility of our NV platform was further demonstrated by its capacity to induce robust mucosal immunity following intranasal booster immunization. This route triggered significant CD4^+^ and CD8^+^ T_RM_, high mucosal IgA levels, and robust systemic immunity. These findings underscore the potential of our NV platform not only for SARS‐CoV‐2 but also for other pathogens requiring mucosal immunity.

Importantly, our study achieved robust immunogenicity using NV based on the biocompatible and biodegradable PLGA/PLA polymeric products approved for human use, manufactured using industrial‐scale methodology ensuring high batch‐to‐batch reproducibility and suitability for continuous manufacturing. These methods reduce the labor intensity and time consumption associated with batch processing.

Our thermostable NV can be stored as a powder, significantly reducing storage and distribution costs and extending shelf‐life, making logistics more feasible and affordable, particularly in remote or resource‐limited areas, such as low‐ and middle‐income countries.

In conclusion, our comprehensive epitope selection approach and innovative delivery system underscore the potential of our NV platform to elicit broad and durable immune responses against SARS‐CoV‐2. Incorporation of siRNA targeting immune checkpoints further enhances the vaccine efficacy, presenting a promising strategy for next‐generation vaccines. This study provides important insights for optimizing the NV platform for other infectious diseases such as HIV, respiratory syncytial virus, Zika, and Rabies. By leveraging bioinformatics prediction tools alongside our “plug and play” polymeric‐based nanoplatform, we can be one step ahead of future pandemics.

## 8 Experimental Section

### Material and Reagents

PLA (2,000 Da) with a weight‐averaged molecular mass (Mw) of 2000 was purchased from PolySciences, Inc. PLGA Resomer RG 503H with a Mw range 24000 – 38000 was purchased from Sigma, poly(vinyl alcohol) (PVA, Mw 13000 – 23,000 Da) was purchased from Sigma, dichloromethane (DCM), (deuterated) dimethyl sulfoxide (DMSO or dDMSO), dimethylformamide (DMF), 4‐dimethylaminopyridine (DMAP), D‐mannosamine hydrochloride, fluorescamine, paraformaldehyde (PFA) 4% (v/v), TPGS, Corning High binding 96 and 384 well plates, tetramethylbenzidine (TMB) ultra‐sensitive, blue, horseradish peroxidase substrate D‐(+)‐Trehalose dihydrate, and bovine serum albumin (BSA) were purchased from Sigma‐Aldrich. N‐butyl poly‐L‐arginine hydrochloride (pARG, Mw range 3000 – 3400) was purchased from Polypeptide Therapeutic Solutions. Phosphate buffered saline (PBS, pH 7.4), Quant‐iT RNA Assay Kit (broad range), Quant‐iT OliGreen ssDNA assay kit, HEPES buffer (1 M), β‐mercaptoethanol (50 mM), LIVE/DEAD fixable yellow dead cell stain kit (for 405 nm excitation), ACK lysing buffer and CD28 Monoclonal Antibody (37.51), eBioscience were purchased from Thermo Fisher Scientific. SARS‐CoV‐2 antigens (Table S1, Supporting Information) were purchased from GeneCust, ProteoGenix SAS, or Sigma–Aldrich. Collagenase type II, and DNase I were purchased from Worthington Biochemical Corporation. CpG‐ODN 1826 (TCCATGACGTTCCTGACGTT) and small interfering RNA (siRNA) anti‐PD‐L1 were purchased from Merck. Poly(I:C) (High Mw) VacciGrade was purchased from InvivoGen. Fluorochrome‐labeled antibodies, permeabilization 10x, and intracellular fixation buffer were purchased from BioLegend, Miltenyi, Biogems, and Thermo Fisher. ELISpot kit was purchased from R&D Systems Inc. Peroxidase AffiniPure Goat Anti‐Mouse IgG and IgM from Jackson Immuno Research Laboratories. RBD protein was produced as reported.^[^ ^45^ ^]^ RBD variants were purchased from ProteoGenix SAS. Human ACE2 was purchased from InvivoGen.

### Computational Methods

Prediction of B‐cell and T‐cell epitopes of the SARS‐CoV‐2 Spike protein was done using various predictions (summarized by Figure S1, Supporting Information). HLA types were selected based on their estimated global population coverage.^[^ ^46^ ^]^ B cell epitope prediction methods that use PDB structures were used with SARS‐CoV‐2 spike ectodomain structure in the open state, PDB ID 6VYB chain B.^[^ ^47^ ^]^ (1) Conservation analysis was performed using ConSurf server,^[^ ^48^ ^]^ using 2288 non‐redundant SARS‐CoV‐2 spike sequences collected from NCBI Virus database on May 19^th^ 2020, and aligned using Mafft.^[^ ^49^ ^]^ (2) Epitope predictions methods: a. MHC‐I: NetMHC for HLA‐B*27:05,^[^ ^50^ ^]^ IEDB MHC‐I (HLA types of HLA‐B*27:05, HLA‐B*58:01, HLA‐B*18:01 and HLA‐C*15:02),^[^ ^51^ ^]^ SYFPEITHI MHC‐I (HLA types HLA‐B*27:05, HLA‐B*18:01),^[^ ^52^ ^]^ EpiJen for HLA‐B*27:05.^[^ ^53^ ^]^ b. MHC‐II for HLA‐DRB1*0101: SYFPEITHI,^[^ ^52^ ^]^ MHCpred,^[^ ^54^ ^]^ and RANKPEP.^[^ ^55^ ^]^ c. B cell epitopes: ABCpred,^[^ ^56^ ^]^ BepiPred,^[^ ^57^ ^]^ ElliPro,^[^ ^58^ ^]^ DiscoTope 2.0,^[^ ^59^ ^]^ SEPPA3, using PDB structures 6VYB,^[^ ^60^ ^]^ EPCES using PDB structure 6VYB.^[^ ^61^ ^]^ (3) Domains annotation was taken from Uniprot.^[^ ^62^ ^]^ (4) Relative surface accessible surface area (SASA) for PDB 6VYB chain B was calculated using the protein structure and interaction analyzer^[^ ^63^ ^]^ with a probe radius of 1.4 Å. (5) Glycosylation sites were taken from Yasunori et al.^[^ ^64^ ^]^ (6) B‐cell immunodominant regions based on SARS‐specific epitope mapping, Dominant SARS‐CoV T‐cell epitopes (100% identical to SARS‐CoV‐2), and Dominant SARS‐CoV B cell epitope regions were taken from Grifoni et al.^[^ ^65^ ^]^

All methods were projected along with spike protein sequence (NCBI protein YP_009724390.1) using JalView.^[^ ^66^ ^]^

The above methods were combined to select the best peptides. Hydrophobicity was predicted using the Thermo Fisher Peptide Analyzing Tool. Peptide 14 hydrophobicity score was 27.83, and peptide 15 was 43.11.

### Epitope Mapping by ELISA

Corning High binding 96 well plates were precoated with peptides (10 µg ml^−1^) overnight at 4 °C in carbonate buffer (pH 9.6). Plates were washed three times with PBS‐T (PBS+0.05% Tween‐20) and blocked with 1% Casein (Sigma‐Aldrich) PBS‐T (PBS+0.1% Tween‐20) for 1.5 h at 37 °C. After three washes with PBS‐T, plates were incubated with plasma samples diluted in PBS‐T (PBS+0.05% Tween‐20) with 1% BSA at a concentration of 10 nm. After 1 h incubation at 24 °C, plates were washed and incubated with goat anti‐human IgG Fc horseradish peroxidase (HRP) conjugated (Abcam) at a 1:20000 dilution. Following 1 h incubation at 24 °C, plates were washed, and the signal was developed using TMB ultra‐sensitive, blue, horseradish peroxidase substrate (Sigma‐Aldrich). The reaction was stopped 10 min later by adding 0.5 m sulfuric acid to wells and the absorbance read at 450 nm. The binding epitope data was submitted to the ClustVis software. Data were pre‐processed by applying a new variance scale and correlations between variables were performed through Pearson's rank test.

### Synthesis of NP

NP was formulated by the double emulsion–solvent evaporation method, following methods already established.^[^ ^23^ ^]^ A man‐PLGA/PLA (2:8) blend was dissolved in DCM at 50 mg ml^−1^. A 10% (m/v) PVA aqueous solution (100 µl) containing CpG at 0.5 mg ml^−1^, Poly(I:C) at 1.0 mg ml^−1^, and SARS‐CoV‐2 antigens (Table S1, Supporting Information) at 10 mg ml^−1^ was added to DCM. A 10% (m/v) PVA aqueous solution was added for an empty NP. The mixture was emulsified with a microprobe ultrasonic processor for 15 s at 20% amplitude. A 2.5% (m/v) TPGS aqueous solution (400 µl) was added, and the second emulsion was formed using the same conditions. The double emulsion was added dropwise into a 0.125% (m/v) PF‐127 aqueous solution and stirred for 1 h at RT. Particle suspension was collected by centrifugation at 20,000 g for 45 min, 4 °C (Beckman J2‐21 m/E High‐Speed Centrifuge). Particles were washed with ultrapure water, collected by centrifugation, and finally resuspended in PBS or ultrapure water. Similarly, siRNA NP was formulated by the double emulsion–solvent evaporation method, following the above method with a prior step of *N*‐butyl‐poly‐*L*‐arginine cationic polymer complexed with siRNA. In short, 12.5 µg of siRNA was complexed with *N*‐butyl‐poly‐*L*‐arginine cationic polymer, in RNase‐free ultra‐pure water. Next, the complex was encapsulated with MHC‐I and MHC‐II peptide and adjuvants as described above to reach a total of 25 µg mouse^−1^.

### Size Distribution and ζ Potential Measurements

Particle size and polydispersity index (Ð) were determined by dynamic light scattering using the Zetasizer Nano ZS equipment (Malvern Instruments). The particle ζ potential was measured by laser Doppler velocimetry in combination with phase analysis light scattering with the same equipment. Particles were diluted in ultrapure water and the electrophoretic mobility was determined at 25 °C with the Helmholtz–Smoluchowski model by cumulative analysis. In addition, the size, PD‐index, and ζ potential of the lyophilized NP were measured by dynamic light scattering (DLS) (Wyatt technology). In short, NP were re‐suspended in 5% D‐(+)‐Trehalose dihydrate (w/v in UPW) and lyophilized in a BenchTop Freeze Dryer (SP Scientific VirTis AdVantage 2.0).

### Particle Morphology by Atomic Force Microscopy (AFM)

Particles were diluted at 10 mg ml^−1^ in ultrapure water. A drop of the sample was placed onto freshly cleaved mica for 20 min and dried with pure nitrogen. Samples were analyzed in tapping mode in the air at RT using a Nanoscope IIIa Multimode (Digital Instruments/Veeco) atomic force microscope and etched silicon tips (≈300 kHz) at a scan rate of ≈1.6 Hz.

### Scanning Electron Microscopy

Particles were diluted in trehalose 5% (m/v) and fast frozen at −80 °C for 2 h. Samples were dried under vacuum, first at −20 °C for 14 h and then at 20 °C for 2 h. Dried specimens were coated with gold on a Peltier cold‐stage sputter coater and examined using a FEI Quanta 200 FEG ESEM Phillips 500 scanning electron microscope at a 5 kV accelerating voltage.

### Transmission Electron Microscopy (TEM)

Particles were diluted in PBS, placed on a carbon‐coated copper grid, and dried. The samples were analyzed with a Philips CM 120 Bio‐Twin transmission electron microscope.

### NP Internalization into BMDC

To test the NP internalization in vitro, we isolated hematopoietic stem cells from the bone marrow of C57BL/6J mice.^[^ ^67^ ^]^ C57BL/6J mice were euthanized, and the bones of the hind limbs were fully removed. Bone marrow cells were extracted by rinsing the bone cavity with RPMI (Thermo Fisher Scientific) medium using a 25G needle. The cellular suspension was filtered by a 70 µm cell strainer, and red blood cell (RBC) lysis was performed (RBC lysis, BioLegend). Finally, cells were suspended in RPMI supplemented with 10% (v/v) FBS, 1% (v/v) PEST, 1% (v/v) HEPES, 1% (v/v) sodium pyruvate, 0.1% (v/v) 2‐Mercaptoethanol and 20 ng ml^−1^ of GM‐CSF Recombinant Mouse Protein. 10^7^/10 ml cells were plated in a low attachment T flask (Sigma–Aldrich) for 7 days. After, clusters of BMDC were lightly bound to a monolayer of tightly adherent fibroblasts. BMDC was harvested, and image stream flow cytometry and flow cytometry assays were performed.

### EE and LC of Antigens and Immune Potentiators

Entrapped SARS‐CoV‐2 antigens and adjuvants were indirectly quantified using the supernatants collected from the centrifugations. The EE (%) (Equation (1)) and LC (µg mg^−1^) (Equation (2)) of SARS‐CoV‐2 antigens were determined using fluorescamine. The relative fluorescence units were measured with a Varioskan Lux Reader (Thermo Fisher) at 382/480 nm for the excitation/emission wavelengths. The amount of Poly(I:C) was determined using the Quant‐iT RNA Assay Kit (broad range), while CpG was determined by the Quant‐iT OliGreen ssDNA Assay Kit, following the manufacturer's instructions. Relative fluorescence units were measured with a Varioskan Lux Reader (Thermo Fisher) at 485/520 excitation/emission wavelengths for binding of OliGreen reagents to CpG and at 644/673 nm excitation/emission wavelengths for RNA Assay kit.

(1) $$\mathrm{EE}\left(\%\right)=\frac{\begin{array}{c} \mathrm{initial}\;\mathrm{amount}\;\mathrm{of}\;\mathrm{biomolecule}\\ \;-\;\mathrm{amount}\;\mathrm{of}\;\mathrm{biomolecule}\;\mathrm{in}\;\mathrm{the}\;\mathrm{supernatant} \end{array}}{\mathrm{initial}\;\mathrm{amount}\;\mathrm{of}\;\mathrm{biomolecule}}\times 100$$

(2) $$\mathrm{LC}\left(\mu g\;{\mathrm{mg}}^{-1}\right)=\frac{\begin{array}{c} \mathrm{initial}\;\mathrm{amount}\;\mathrm{of}\;\mathrm{biomolecule}\\ \;-\;\mathrm{amount}\;\mathrm{of}\;\mathrm{biomolecule}\;\mathrm{in}\;\mathrm{the}\;\mathrm{supernatant} \end{array}}{\mathrm{total}\;\mathrm{amount}\;\mathrm{of}\;\mathrm{polymer}}$$

### Cell Viability Assay

BMDC were obtained as previously described and were seeded in 96 well plates, 10^5^ cells well^−1^. The cells were treated with increasing concentrations of Empty NP (125, 250, 500, 1000 µg ml^−1^), and their viability was tested by cell proliferation kit II (XTT) (Sigma–Aldrich, cat# 11465015001) in several time points (3, 6, 20, 44 h). At the endpoint, cells were incubated with XTT reagent, according to the manufacturer's instructions, for 4 h at 37 °C, and sample optical density (OD) was measured by a SpectraMax plate reader (Molecular Devices) at 450 nm.

### Animal Studies

All the animal procedures were performed in compliance with the Portuguese competent authority for animal protection (Direcção Geral de Alimentação e Veterinária) and Sackler Faculty of Medicine, Tel Aviv University guidelines. The protocols (0421/000/000/2021 and 01‐20‐060) were approved by the Institutional Animal Care and Use Committees at the University of Lisbon or at Tel Aviv University and performed per National Institutes of Health guidelines. Male C57BL/6J mice (8‐week‐ old) were purchased from Instituto Gulbenkian de Ciência (IGC) or Envigo Ltd. and housed in the animal facility of the Faculty of Pharmacy, University of Lisbon or at Tel Aviv University. Mouse body weight change was monitored three times per week until day 28 after 1^st^ immunization and two times per week after (Figure S5C, Supporting Information). Mice were euthanized according to ethical protocols.

### In Vivo Safety

All behavioral studies, open field, and RotaRod, were conducted in the Myers Neuro‐Behavioral Core Facility (Tel‐Aviv University, Israel). C57BL/6J male mice (8‐week‐old) were randomized into eight groups (*N* = 10). The treatment (NV‐8‐ Figure 2, or siPD‐L1 NV‐8‐ Figure S9, Supporting Information, a total of 400 µg peptide mouse^−1^) was administered SC or IN in two doses (prime and boost), one week apart. To detect the behavioral change, mice were tested before and after each treatment. **Open‐field**: The open field consisted of a 50 × 50×40 cm plexiglass arena with a white floor and light intensity of 300 lx. Each mouse was placed in a corner of the arena and allowed free exploration of the arena for 15 min. Mouse behavior was continuously recorded by a video camera placed over the structure and analyzed using EthoVision‐XT software (Noldus Information Technology). **RotaRod**: Mice were placed on a 5‐lane accelerating Rotarod (Ugo Basile, Italy) for balance assessment. Each mouse was placed on a 3‐cm‐diameter horizontal rod elevated 16 cm from the ground. Mice were subjected to five trials in every session, of which the three highest‐score test trials were averaged. A trial begins with the rod spinning at 4 RPM and gradually accelerating by a factor of 0.5 cm s^−1^ every 5 s to a maximum of 50 RPM. The latency until falling from the rod was measured and analyzed. One week post the second NV injection, following the RotaRod and open‐field tests, mice were euthanized and blood was collected to assess the effect of NV treatment on mice blood chemistry and blood count in vivo. Blood samples were analyzed by American Medical Laboratories (AML) Ltd (Herzliya, Israel). Additionally, the nasal mucosa morphology was visualized by hematoxylin and eosin (H&E) in mice treated twice IN (Figure S9, Supporting Information). One week after the final nasal vaccination, the nasal tissues were excised and fixed for 48 h with 4% paraformaldehyde (PFA) in PBS (CAS# 30525‐89‐4, Thermo Scientific) at 4 °C. Then, the tissues were transferred into 0.5 M EDTA (CAS# 6381‐92‐6, Sigma) solution (the pH was adjusted with NaOH to reach pH7) for 24 h at RT. Next, the tissue was embedded in paraffin and cut into 8 µm sections. The coronal nasal sections were stained with H&E by Leica ST5020‐CV5030 Stainer and observed using BioTek Cytation C10 Confocal Imaging Reader (Agilent Technologies, Inc.) by 60x objective. The sections were analyzed morphologically to detect any damage to the nasal mucosa tissue induced by the nasal administration of our treatments.

### Immunization of Mice with SARS‐CoV‐2 Antigens

For immunization studies, 8‐week‐old C57BL/6J male mice were randomized into treatment groups (*N* = 8–15). For SC immunization, NV (100 µl) was injected into each mouse proximal to popliteal lymph nodes. A half dose (50 µl) was injected into the right side and the other half into the left side. For IN immunization, NV (30 µl) was administered into each mouse nostril. A half dose (15 µl) was injected into the right nostril and the other half into the left. Each dose contained 400 µg of antigen (200 µg of P14 and 200 µg of P15) plus 20 µg of CpG and 40 µg of Poly(I:C), either free in solution or entrapped in 2 mg of particles (20 mg ml^−1^).

Moreover, we performed a comparison with the commercially available Pfizer‐BioNTech COVID‐19 Vaccine (also known as COMIRNATY), obtained from Dr. Armando Alcobia (Hospital Garcia d'Orta). This vaccine was diluted in sterile PBS and 50 µl containing 1 µg was injected into the mice.

### ELISA

ELISA was performed for the detection of peptides (Table S1, Supporting Information) or SARS‐CoV‐2 RBD‐specific antibodies in immunized mouse sera. Corning High binding 96‐well plates were precoated with peptides (10 µg ml^−1^) or RBD protein (1 µg ml^−1^) overnight at 4 °C in carbonate buffer (pH 9.6). Plates were washed three times with PBS‐T (PBS+0.05% Tween‐20) and blocked with 3% BSA (Sigma‐Aldrich, #A8022) in PBS‐T (PBS+0.1% Tween‐20) for 2 h at 37 °C. After three washes with PBS‐T, plates were incubated with serially diluted mouse sera in PBS‐T/1% BSA for 1 h at 24 °C. Following washing, Peroxidase AffiniPure Goat Anti‐Mouse IgG, IgM (Jackson Immuno Research Laboratories), IgG2a and IgG1 (Southernbiotec), or IgA Cross‐Adsorbed Secondary Antibody, HRP (ThermoFisher) were added for 1 h at 24 °C. The plates were washed with PBS‐T and reactions were developed with TMB ultra‐sensitive, blue, horseradish peroxidase substrate (Sigma‐Aldrich). The reaction was stopped by adding 0.5 M of sulfuric acid. Plates were read at 405 nm absorbance using the Varioskan Lux Reader (Thermo Fisher). Antibody titers were calculated as the highest serum dilution with an OD value above 2 times the average OD of the negative control.

### Cellular Immune Responses Assessed by Flow Cytometry

On day 28, mice were euthanized, the spleens harvested and the splenocytes isolated. Splenocytes from each group were seeded at 3–4 × 10^6^ cells per in‐well at 6‐well plates. Splenocytes were cultured with 100 µg.ml^−1^ of P14 and P15 and 2 µg ml^−1^ of CD28 or medium only (negative control). After incubation at 37 °C for 6 h in the presence of Brefeldin for the last 4 h of culturing, cells were labeled for surface markers (CD3, CD4, CD8, CD25, CD107b, and CD127 [Biolegend]) and the LIVE/DEAD Yellow indicator dye (Life Technologies) was added. The intracellular cytokines were detected by antibodies specific for Th1 cytokines IFN‐γ, TNF‐α, and IL−2; Th2 cytokines IL‐4, IL6 and IL‐10 (Biolegend). The samples were processed using the Cytek Aurora flow cytometer (Cytek). Data were analyzed using FlowJo (BD Bioscience) using the presented gating strategy (Figure S10, Supporting Information).

### Functional Assessment of T Cells

For the ELISpot assay, on day 0 mice were randomized into 4 groups, and treated according to the schedule used in Figure 3. On day 28, mice were euthanized, the spleens harvested and the splenocytes isolated. Splenocytes were seeded at 2 × 10^5^ cells per well in 96‐well plates coated with IFN‐γ antibody (R&D Systems Inc.) and incubated for 20 h with 2 µg ml^−1^ of CD28 (Invitrogen) and 1 mg ml^−1^ of peptides 14 and 15. The secreted and captured IFN‐γ was subsequently detected using a biotinylated antibody specific for IFN‐γ and an alkaline‐phosphatase conjugated to streptavidin. After the addition of the substrate solution, a blue precipitate formed and appeared as spots at the sites of cytokine localization. Automated spot quantification was performed using the Cytation 7 (Biotek).

### Immune Profiling of Nasal Mucosa by Flow Cytometry

On day 28, mice were euthanized, and the cells from nasal turbinates were isolated. Briefly, nasal turbinates were minced with scissors, incubated in a digestion cocktail containing collagenase type II and DNase I in RPMI at 37 °C for 45 min, and dissociated through a 70‐µm filter. Cells were treated with ammonium‐chloride‐potassium (ACK) buffer to lyse red blood cells and then washed once with PBS. Single‐cell suspensions were incubated with LIVE/DEAD Yellow indicator dye (Life Technologies) and anti‐mouse TruStain FcX PLUS CD16/32 (Biolegend) for 20 min at 4 °C. Cells were washed once with PBS before surface staining. T cells were stained for CD3, CD44, CD4, CD8, CD69, and CD103 for 20 min at 4 °C. For B cell analysis, cells were stained for GL7, IgM, CD138, CD19, IgA, B220, CD38, CD138, and IgD for 20 min at 4 °C. Cells were washed with PBS once, followed by 4% paraformaldehyde fixation for 20 min at 4 °C. The samples were acquired using the Cytek Aurora flow cytometer (Cytek). Data were analyzed using FCS Express (De Novo Software) using the presented gating strategy (Figure S11, Supporting Information).

### NV‐Induced Memory T Cell Assessment

On day 95, all mice groups were challenged with an IV injection of 200 µg of peptide 14, 200 µg of peptide 15, 20 µg of CpG, and 40 µg of Poly(I:C), free in PBS. Blood was collected from mice's cheeks on days 91 and 101 for antibody detection by ELISA. On day 101, mice were euthanized, the spleens harvested, and the splenocytes isolated. Splenocytes from each group were labeled for surface markers [CD8, CD3, CD4, CD69, CD44, CD62L, CD38 (Miltenyi), IgG (BioLegend), B220 (Biogems)] and the Zombie LIVE/DEAD indicator dye (Invitrogen) was added. The samples were processed using the Cytek Aurora flow cytometer (Cytek). Data were analyzed using SpectroFlo.Ink software.

### Quantitative Real‐Time RT‐PCR

Frozen spleen samples were homogenized using a motor‐driven grinder on TRIzol reagent (Invitrogen, Thermo Fisher Scientific, Waltham, MA, USA), and then total RNA was extracted following the manufacturer's instructions. Total RNA was quantified by Qubit 2.0 fluorometer (Invitrogen, Thermo Fisher Scientific), and 1.5 µg RNA was converted into cDNA using NZY First‐Strand cDNA Synthesis Kit (NZYTech, Lisbon, Portugal), according to the manufacturer's protocol. Quantitative real‐time RT‐PCR (qPCR) was performed using QuantStudio 7 Flex Real‐Time PCR System (Applied Biosystems, Thermo Fisher Scientific). qPCR was performed in 5 µl duplicate reactions on a 384‐well QuantStudio 7 Flex Real‐Time PCR System (Applied Biosystems, Thermo Fisher Scientific), using the 2x SensiFAST SYBR Hi‐ROX kit (Bioline, Meridian Bioscience, Inc., Cincinnati, OH, USA), following manufacturer's protocol. The following primer sequences were used: for Pd‐l1 gene (Cd274) 5′ ATT CTC TGG TTG ATT TTG CGG TA 3`(forward) and 5′ TTC AGA TCA CAG ACG TCA AGC TG 3′ (reverse); for the hypoxanthine phosphoribosyltransferase (Hprt) gene, 5′ GGT GAA AAG GAC CTC TCG AAG TG 3′ (forward) and 5′ ATA GTC AAG GGC ATA TCC AAC AAC A 3′ (reverse). The relative amount of Pd‐l1 was calculated based on the standard curve and was normalized to the level of Hprt, being expressed as fold change from PBS controls.

### sVNT Assay

A Corning High binding 96‐well plate was precoated with WT RBD protein or RBD variants (1 µg ml^−1^) overnight at 4 °C in carbonate buffer (pH 9.6). Plates were washed three times with PBS‐T (PBS+0.05% Tween‐20) and blocked with 3% BSA (Sigma‐Aldrich, #A8022) in PBS‐T (PBS+0.1% Tween‐20) for 2 h at 37 °C. After three washes with PBS‐T, plates were incubated with ACE2‐biotin (InvivoGen) and mouse sera (1:2) (final volume of 25 µl) in PBS‐T/1% BSA for 1 h at 24 °C. Following washing, streptavidin‐HRP (Sigma‐Aldrich) was added for 1 h at 24 °C. The plates were washed with PBS‐T and reactions were developed with TMB ultra‐sensitive, blue, horseradish peroxidase substrate (Sigma‐Aldrich). The reaction was stopped by adding 0.5 M of sulfuric acid. The absorbance readings at 405 nm were acquired using the Varioskan Lux Reader (ThermoFisher Scientific). The OD values were converted to a common scale of 0–100. Inhibition (%) was measured through the following metrics: [1 – (OD value of unknown sample/OD value of Max interaction) x 100.

NT_50_ calculation: Levels of 50% neutralizing titer (NT_50_) were determined using log10 (serum dilution) versus the normalized neutralization function of GraphPad Prism software (V10.1.1).

### SARS‐CoV‐2 Infection and Treatment in Mice

Animal housing and experimental procedures were conducted according to the French and European Regulations (Parlement Européen et du Conseil du 22 Septembre 2010, Decret n° 2013–118 du 1er fevrier 2013 relatif à la protection des animaux utilisées a des fins scientifiques) and the National Research Council Guide for the Care and Use of Laboratory Animals (National Research Council (U. S.), Institute for Laboratory Animal Research (U.S.), and National Academies Press (U.S.), Eds., Guide for the care and use of laboratory animals, 8^th^ ed. Washington, D.C: National Academies Press, 2011). The animal BSL3 facility is authorized by the French authorities (Agreement N° B 13 014 07). All animal procedures (including surgery, anesthesia, and euthanasia, as applicable) used in the current study were submitted to the Institutional Animal Care and Use Committee of the CIPHE and approved by the French authorities (APAFIS#26484‐2020062213431976 v6). All CIPHE BSL3 facility operations are overseen by a biosecurity/biosafety officer and accredited by the Agence Nationale de Sécuritée du Médicament (ANSM).

Heterozygous K18‐hACE C57BL/6J mice (strain: 2B6. Cg‐Tg (K18‐ACE2)2Prlmn/J) were obtained from The Jackson Laboratory. All breeding, genotyping, and production of K18‐hACE2 was performed at the CIPHE under specific pathogen‐free conditions and following animal care and use regulations. Mice were housed under a 12 h dark:12 h light cycle, with a temperature range of 20–22 °C and a humidity range of 40–70%. The sample size was based on previous articles reporting the use of K18‐hACE2 mice in SARS‐CoV‐2 challenge experiments (10 animals per experimental group). Animals were housed in groups within cages and fed with standard chow diets. All animals used were age‐matched females.

### Wuhan/D614 SARS‐CoV‐2 Virus Production

Vero E6 cells (CRL‐1586; American Type Culture Collection) were cultured at 37 °C in Dulbecco's modified Eagle's medium (DMEM) supplemented with 10% fetal bovine serum (FBS), 10 mM HEPES (pH 7.3), 1 mM sodium pyruvate, 1X non‐essential amino acids, and 100 U mL^−1^ penicillin/streptomycin. The strain Beta CoV/France/IDF0372/2020 was supplied by the National Reference Centre for Respiratory Viruses hosted by the Institut Pasteur (Paris, France). The human sample from which strain BetaCoV/France/IDF0372/2020 was isolated was provided by the Bichat Hospital, Paris, France. Infectious stocks were grown by inoculating Vero E6 cells and collecting supernatants upon observation of the cytopathic effect. Debris was removed by centrifugation and passage through a 0.22 mm filter. Supernatants were stored at 80 °C.

### Immunization and SARS‐CoV‐2 Infection Challenge Assay in K18‐hACE2 Transgenic Mice

Eight to twelve‐week‐old heterozygous K18‐hACE2 mice (*N* = 10 or 11) received a subcutaneous prime immunization with siPD‐L1 NV‐8. Mice received the subcutaneous or intranasal booster 3 weeks later. Two weeks after the boost immunization, mice were infected intranasally with 10^5^ PFU SARS‐CoV‐2 via intranasal administration of 30 µL. Intranasal virus treatment was performed under anesthesia, and all efforts were made to minimize animal suffering. Mice were monitored daily for morbidity (body weight) and mortality (survival). During the monitoring period, mice were scored for clinical symptoms (weight loss, eye closure, appearance of the fur, posture, and respiration). Mice obtaining a clinical score defined as reaching the experimental endpoint were humanely euthanized.

### Measurement of SARS‐CoV‐2 Viral Load TCID_50_ (50% of Tissue‐Culture Infective Dose)

The median tissue‐culture infectious dose (TCID_50_) represents the dilution of a virus‐containing sample in which half of the inoculated cells show signs of infection. To perform the assay, lung, and brain tissues were weighed and homogenized using ceramic beads in a tissue homogenizer (Precellys Bertin Instruments) in 0.5 mL RPMI media supplemented with 2% FCS and 25 mM HEPES. Tissue homogenates were then clarified by centrifugation and stored at 80 °C until use. Forty‐thousand cells per well were seeded in 96‐well plates containing 200 µL DMEM +4% FCS and incubated for 24 h at 37 °C. Tissue homogenates were serially diluted (1:10) in RPMI media, and 50 µL of each dilution was transferred to the plate in six replicates for titration at five days post‐inoculation. Plates were read for the CPE (cytopathology effect) using a microscopy reader, and the data were recorded. Viral titers were then calculated using the Spearman‐Karber formula and expressed as TCID_50_/mg of tissue.

### Cytokine and Chemokine Protein Measurements

On day 5 post‐infection, blood was collected, and plasma was isolated after centrifugation. The lung and brain were harvested in Precellys tubes containing RPMI medium completed with Pen/Strep, HEPES, and FCS, and then homogenized with the Precellys. Then the samples were inactivated by a mix of Triton 10X and RPMI medium at a final concentration of 0.5% Triton. A kit, created by Merck Millipore, to cover the overall cytokine and chemokine responses was used here. This kit, coupled with the Luminex platform in a magnetic bead format, provides the advantage of ideal speed and sensitivity, allowing quantitative multiplex detection of dozens of analytes simultaneously, which can dramatically improve productivity. It contained cytokines standards to make a standard curve and to ensure lot‐to‐lot consistency, two quality controls to qualify assay performance, detection antibody cocktails designed to yield consistent analyte profiles within the panel, streptavidin‐PE and a panel of magnetic beads that recognize each one of the following analytes, IL‐1β and IFN‐γ. In a 96‐well plate previously washed with wash buffer, we incubated overnight (at 4 °C and on a shaker) and according to a template, the standards, the quality controls, and the samples (plasma and organs supernatants) with assay buffer and magnetic beads panel. The following day the plate was washed twice with wash buffer, then we added the detection antibodies cocktail and incubated 1 h at RT on the shaker before we added the Streptavidin‐PE and incubated for 30 min. Then, the plate was washed twice, and the samples were suspended in an assay buffer for 5 min on a shaker before reading on MagPix Instrument.

## Conflict of Interest

R.S.‐F. is a Board Director at Teva Pharmaceutical Industries, Ltd. All other authors declare that they have no competing interests.

## Supporting information

Supporting Information

## Data Availability

The data that support the findings of this study are available in the supplementary material of this article.
