# Supplementary material for: Intranasal Multiepitope PD‐L1‐siRNA‐Based Nanovaccine: The Next‐Gen COVID‐19 Immunotherapy
Source: Adv Sci (Weinh). 2024 Aug 8;11(40):2404159. doi: 10.1002/advs.202404159 (PMC11515909; doi:10.1002/advs.202404159)
Supplement: Supplementary file 1 — Supporting Information [file ADVS-11-2404159-s001.docx]

**Supplementary Information**


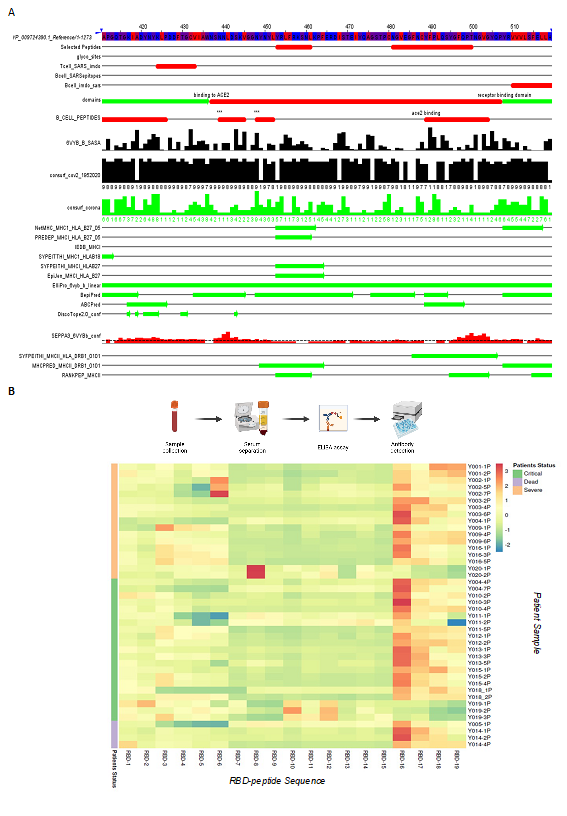


**Figure S1. SARS-CoV-2 peptide selection immunoinformatic analysis workflow. A)** Bioinformatic analysis of the S protein. The reference sequence^23^ is colored by the Kyte-Doolitle hydrophobicity scale. Only residues 411-519 are shown for clarity. The selected peptides 14 and 15 are shown in red lines under the sequence. No glycosylation sites were predicted for this area. Known T-cell and B-cell epitopes from SARS-CoV are also shown. The two RBD peptides are both included within the ACE2 binding region. Solvent accessibility surface area, calculated using PDB ID 6VYB, chain B, is shown by black bars, with higher bars marking higher water accessibility. Conservation among other SARS-Cov-2 sequences, collected until 19/5/2020 is shown by black bars, with higher bars marking a high conservation score. Conservation among other corona family sequences is shown by green bars. Epitope prediction methods are indicated by green arrows. SEPPA3 B-cell epitope prediction is shown by red bars, with a 0.064 threshold dashed black line. **B)** RBD-epitope mapping. The results are summarized as a heatmap. In total, plasma samples of 42 COVID-19 patients were used to evaluate peptide reactivity.

**Table S1. SARS-CoV-2 peptide antigens.** The SARS-CoV-2 peptide sequences are paired according to the vaccine candidates and SARS-CoV-2 structural proteins to which they belong.

| **NV** | **SARS-CoV-2 Region** | **Peptide Sequence** | **#** | **MHCI** | **MHC II** | **Epitope** | |
| --- | --- | --- | --- | --- | --- | --- | --- |
|  |  |  |  |  |  | **T cell** | **B cell** |
| 1 | S (327-335) | VRFPNITNL | 1 | √ | - | √ | - |
|  | S (802-819) | FSQILPDPSKPSKRSFIE | 2 | - | √ | - | √ |
| 2 | N (322-331) | MEVTPSGTWL | 3 | √ | - | √ | - |
|  | N (168-182) | PKGFYAEGSRGGSQA | 4 | - | √ | √ | √ |
| 3 | S (996-1004) | LITGRLQSL | 5 | √ | - | √ | √ |
|  | S (891-904) | GAALQIPFAMQMAY | 6 | - | √ | √ | √ |
| 4 | S (820-829) | DLLFNKVTLA | 7 | √ | - | √ | - |
|  | S (389-403) | DLCFTNVYADSFVIR | 8 | - | √ | √ | √ |
| 5 | N (96-105) | GGDGKMKDLS | 9 | √ | - | √ | - |
|  | N (156-170) | AIVLQLPQGTTLPKG | 10 | - | √ | √ | - |
| 6 | M (198-206) | RYRIGNYKL | 11 | √ | - | √ | - |
|  | M (176-190) | LSYYKLGASQRVAGD | 12 | - | √ | √ | - |
| 7 | S (489-504) | FPLQSYGFQPTNGVG | 13 | - | √ | √ | - |
|  | S (453-461) | YRLFRKSNL | 14 | √ | - | √ | - |
| 8 | S (453-461) | YRLFRKSNL | 14 | √ | - | √ | - |
|  | S (481-500) | NGVEGFNCYFPLQSYGFQPT | 15 | - | √ | √ | √ |
| 9 | S (491-500) | PLQSYGFQPT | 16 | √ | - | √ | - |
|  | S (491-500) | NGVEGFNCYFPLQSYGFQPT | 15 | - | √ | √ | √ |
| 10 | S (481-490) | NGVEGFNCYF | 17 | √ | - | √ | - |
|  | S (481-500) | NGVEGFNCYFPLQSYGFQPT | 15 | - | √ | √ | √ |
| 11 | S (484-493) | EGFNCYFPLQ | 18 | √ | - | √ | - |
|  | S (481-500) | NGVEGFNCYFPLQSYGFQPT | 15 | - | √ | √ | √ |
| NV- Nanovaccine Candidate; MHC- Major Histocompatibility Complex; S- Spike Protein; N- Nucleocapsid Protein; M-Membrane Protein. | | | | | | | |

**Table S2.** Amino acid sequence of the synthesized peptides used for epitope mapping and their location in the Wuhan-Hu-1 S protein sequence. In green the MHC-II peptide 16 was used on vaccine candidates NV-8 to NV-12.

| **Peptide** | **Position in Spike sequence** | **Amino acid sequence** |
| --- | --- | --- |
| **RBD-1** | (331-350) | NITNLCPFGEVFNATRFASV |
| **RBD-2** | (341-360) | VFNATRFASVYAWNRKRISN |
| **RBD-3** | (351-370) | YAWNRKRISNCVADYSVLYN |
| **RBD-4** | (361-380) | CVADYSVLYNSASFSTFKCY |
| **RBD-5** | (371-390) | SASFSTFKCYGVSPTKLNDL |
| **RBD-6** | (381-400) | GVSPTKLNDLCFTNVYADSF |
| **RBD-7** | (391-410) | CFTNVYADSFVIRGDEVRQI |
| **RBD-8** | (401-420) | VIRGDEVRQIAPGQTGKIAD |
| **RBD-9** | (411-430) | APGQTGKIADYNYKLPDDFT |
| **RBD-10** | (421-440) | YNYKLPDDFTGCVIAWNSNN |
| **RBD-11** | (431-450) | GCVIAWNSNNLDSKVGGNYN |
| **RBD-12** | (441-460) | LDSKVGGNYNYLYRLFRKSN |
| **RBD-13** | (451-470) | YLYRLFRKSNLKPFERDIST |
| **RBD-14** | (461-480) | LKPFERDISTEIYQAGSTPC |
| **RBD-15** | (471-490) | EIYQAGSTPCNGVEGFNCYF |
| **RBD-16**  **(P15 in SI Table 1)** | (481-500) | NGVEGFNCYFPLQSYGFQPT |
| **RBD-17** | (491-510) | PLQSYGFQPTNGVGYQPYRV |
| **RBD-18** | (501-520) | NGVGYQPYRVVVLSFELLHA |
| **RBD-19** | (511-524) | VVLSFELLHAPATV |

**Table S3. Nanoparticle (NP) size, polydispersity index (Ð), entrapment efficiency (EE) and loading capacity (LC) of antigens into NP.** The EE and LC of peptides (peptide 1 (P1) to peptide 18 (P18)) were determined by the fluorescamine assay (mean ± s.d.; *N =* 3, *n =* 3).

| **NP** | **Size^1^**  (nm ± s.d.^2^) | Ð **± s.d.**^2^ | **ζ-Potential**  (mV ± s.d.^2^) | **Peptide EE**  (% ± s.d.^2^) | **Peptide LC**  (μg/mg ± s.d.^2^) |
| --- | --- | --- | --- | --- | --- |
| Empty NP | 172 ± 13 | 0.20 ± 0.02 | -8.8 ± 4.3 | NA | NA |
| **P1**-loaded NP | 203 ± 1 | 0.20 ± 0.00 | -0.5 ± 0.14 | 67.5 ± 1.6 | 33.7 ± 0.8 |
| **P2**-loaded NP | 222 ± 8 | 0.20 ± 0.01 | -0.6 ± 0.9 | 70.4 ± 0.3 | 35.2 ± 0.2 |
| **P3**-loaded NP | 172 ± 6 | 0.19 ± 0.01 | -16.1 ± 8.5 | 63.1 ± 2.0 | 31.6 ± 1.0 |
| **P4**-loaded NP | 274 ± 35 | 0.30 ± 0.07 | -16.5 ± 4.6 | 85.4 ± 4.1 | 42.7 ± 2.0 |
| **P5**-loaded NP | 194 ± 6 | 0.22 ± 0.03 | -18.9 ± 6.8 | 66.8 ± 2.0 | 33.4 ± 1.0 |
| **P6**-loaded NP | 176 ± 2 | 0.21 ± 0.01 | -18.9 ± 7.9 | 55.6 ± 2.1 | 27.8 ± 1.0 |
| **P7**-loaded NP | 210 ± 2 | 0.39 ± 0.09 | -12.8 ± 2.9 | 54.7 ± 1.4 | 27.4 ± 0.7 |
| **P8**-loaded NP | 279 ± 11 | 0.47 ± 0.00 | -14.5 ± 4.5 | 60.1 ± 2.9 | 30.0 ± 1.4 |
| **P9**-loaded NP | 167 ± 10 | 0.19 ± 0.03 | -8.2 ± 3.5 | 58.2 ± 2.9 | 29.1 ± 1.4 |
| **P10**-loaded NP | 172 ± 13 | 0.19 ± 0.03 | -10.0 ± 3.4 | 79.5 ± 1.5 | 39.8 ± 0.8 |
| **P11**-loaded NP | 170 ± 5 | 0.18 ± 0.01 | -9.18 ± 3.2 | 75.7 ± 0.9 | 37.8 ± 0.5 |
| **P12**-loaded NP | 170 ± 3 | 0.19 ± 0.02 | -10.1 ± 2.3 | 55.1 ± 3.6 | 27.5 ± 1.8 |
| **P13**-loaded NP | 166 ± 7 | 0.23 ± 0.04 | -9.2 ± 1.0 | 70.6 ± 1.4 | 35.3 ± 0.7 |
| **P14**-loaded NP | 203 ± 1 | 0.15 ± 0.01 | -34.2 ± 1.7 | 74.0 ± 0.5 | 74.0 ± 0.5 |
| **P15**-loaded NP | 188 ± 8 | 0.15 ± 0.06 | -34.4 ± 1.6 | 58.0 ± 6.0 | 58.0 ± 6.0 |
| **P16**-loaded NP | 202 ± 9 | 0.12 ± 0.01 | -34.4 ± 0.9 | 99.5 ± 0.1 | 99.5 ± 0.1 |
| **P17**-loaded NP | 204 ± 2 | 0.14 ± 0.04 | -34.7 ± 1.2 | 84.0 ± 2.1 | 84.0 ± 2.1 |
| **P18**-loaded NP | 190± 6 | 0.12 ± 0.01 | -32.4 ± 0.3 | 78.3 ± 0.4 | 78.3 ± 0.4 |
| NP – Nanoparticle; Ð - Polydispersity index; EE - Entrapment Efficiency; LC - Loading Capacity. The LC calculation differs between P1-13 and P14-19 as the initial amount of peptide was 0.5 mg and 1 mg respectively; NA - Not Applicable. ^1^ Z-average hydrodynamic diameter. ^2^ s.d., standard deviation, obtained from 3 independent batches (*N* = 3). | | | | | |

**Figure S2. NV physicochemical characterization. A-B)** Lyophilized NP stored at 24°C**.** Data represent mean ± s.d. (*n =* 3). The parameters PD Index (Ð) **(A**), and ζ potential (**B)** were measured over 105 days by DLS. **C-D)** Suspension of empty NP and NV in PBS were stored at two temperatures, 4°C and 24°C. PD Index **(C)**, and ζ potential (**D**) were measured over 105 days by DLS, data represent mean ± s.d. (*n =* 5). **E-G)** Suspension of empty NP and NV in PBS were stored at two temperatures, 4°C and 24°C for 3 days. PD Index (**E**), ζ potential (**F**), and hydrodynamic diameter (**G**) were measured by DLS, data represent mean ± s.d. (*n =* 5). **H-I)** Representative images of NP size distribution by DLS: empty NP (**H**) and NV (**I**).


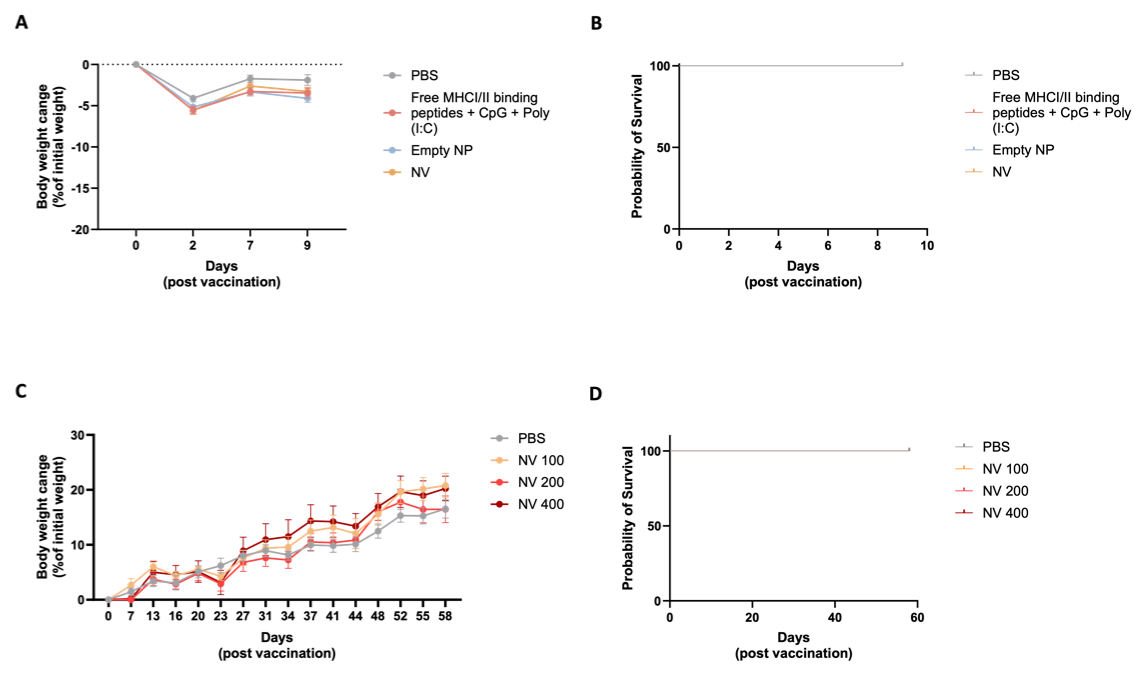


**Figure S3. *In vivo* safety evaluation- NV does not cause a reduction in body weight.** C57BL/6J mice motor function was assessed by RotaRod and open field tests following NV administration. **A)** body weight change, data represent mean ± s.e.m. *N =*10 mice. All group comparisons were NS by the two-way ANOVA test. **B)** Kaplan Meier plot showing the probability of survival, *N =* 10 mice. Escalating NV dose effect on mice safety was done by administrating 3 NV doses (100 mg, 200 mg, and 400 mg of peptide per mouse, addressed as NV 100, NV 200, and NV 400 respectively). **C)** Body weight change, data represent mean ± s.e.m. *N =* 7 mice. All group comparisons were NS by the two-way ANOVA test. **D)** Kaplan Meier plot showing the probability of survival, *N =* 7 mice.

Figure S4. In vivo screening of COVID-19 NV candidates 7-11. A) Immunization scheme of C57BL/6J mice timeline. B) Percentage of T follicular regulatory (T_fr_) cells (CD4+ CXCR5+FOXP3+ PD-1+ cells). C) Frequencies of antigen-specific CD4+ T cells producing T helper 1 (T_H_1) cytokines IFN-γ, TNF-α, and IL-2 evaluated by flow cytometry 1 week after the second NV dose (day 28) and after stimulation with the relevant SARS-CoV-2 peptides for 6 h. Data represents mean ± s.d. N = 3 animals, one-way ANOVA followed by Tukey's multiple comparisons test. D) Percentage of CD3+ CD8+ PD-1+ cells. E-F) SARS-CoV-2 peptide MHCI and MHCII-specific IgG antibodies from blood serum collected on day 35 determined by ELISA (Serum dilution: 1:135 for MHC-I and 1:1215 for MHC-II). Box and whiskers represent the mean, min, and max, N = 5 animals per group, one-way ANOVA followed by Tukey's multiple comparisons test.

**

Figure S5. IgG antibody kinetics of NV-8. A) SARS-CoV-2 RBD-peptide IgG antibodies determined by ELISA for 12 weeks (Serum dilution: 1:1215 peptide 15). B) SARS-CoV-2 RBD-peptides and RBD IgG antibody titers determined by ELISA. Box and whiskers represent the mean, min, and max, one-way ANOVA followed by Tuke’s multiple comparisons test. C) Body weight change of evaluation of NV-8. Data are presented as mean ± s.e.m. N until the endpoint (day 28): 10 mice per group. N after the endpoint: 5 mice per group.

**Figure S6. siPD-L1 NV-8 physicochemical characterization over time. A-B)** Lyophilized empty NP and siPD-L1 NV-8 stored at 24°C**.** Data represent mean ± s.d. (*n =* 3). The parameters Diameter (**A**), and PD Index (Ð) (**B**) were measured over 28 days by DLS. **C-H,** Suspension of empty NP and siPD-L1 NV-8 in PBS were stored at two temperatures, 4°C (**C-E),** and 24°C (**F-H**). Diameter at 4°C (**C**), PD Index at 4°C (**D**), and ζ potential at 4°C (**E**), Diameter at 24°C (**F**), PD Index at 24°C (**G**), and ζ potential at 24°C (**H**) were measured over 28 days by DLS, data represent mean ± s.d. (*n =* 3).

**
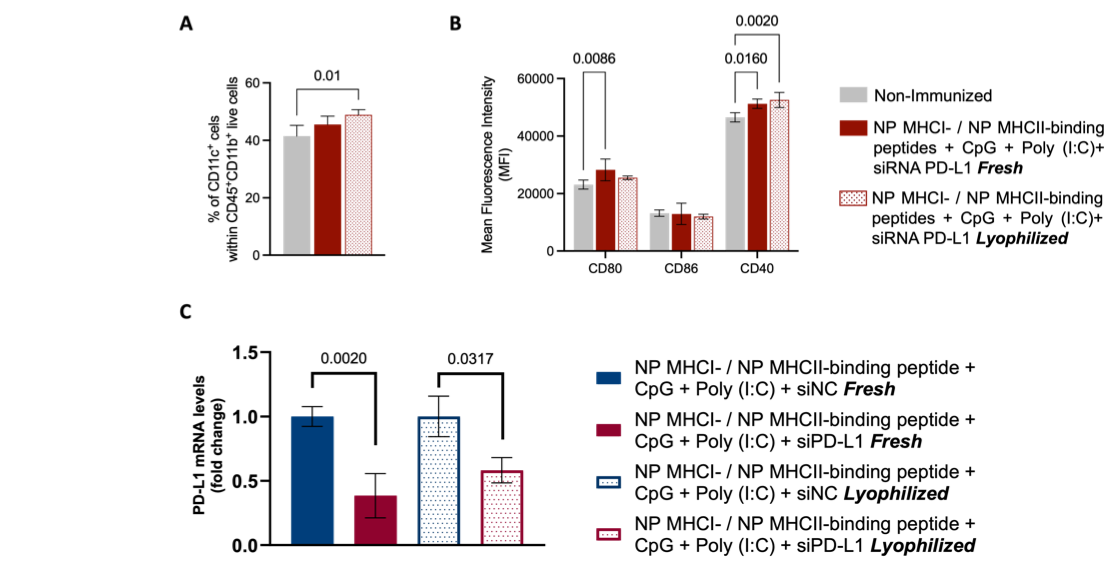
**

**Figure S7.** **NV activity after lyophilization.** After lyophilization, NV was kept at RT until further use. C57BL/6J mice were immunized with lyophilized NV that was reconstituted in PBS or freshly prepared NV. Control mice were not immunized. DC activation was evaluated *in vivo.* DC frequency (**A)** and activation of DC markers (**B**) were assessed 17 hours after immunization. Data represent mean ± s.d. *N* = 4, One-way ANOVA followed by Dunnett’s multiple comparisons test (**A**) and two-way ANOVA followed by Tukey post-hoc test (**B**). **C)** siPD-L1 silencing ability in the I-LN after lyophilization was measured *in vivo* 48 h post SC immunization with NV. mRNA levels were measured by qRT-PCR, while siPD-L1 silencing was compared to a scrambled negative control sequence (NC). Data represent mean ± s.e.m, *N* = 3, one-way ANOVA followed by Tukey post-hoc test.

**Figure S8.** Neutralization antibody titers of SARS-CoV-2 variants. Neutralizing antibody titers (NT_50_) in serum against SARS-CoV-2 RBD Alpha (α) (**A**), Beta (β) (**B**), and Gamma (γ) (**C**) in mice immunized with NV-8, siNC NV-8, and siPD-L1 NV-8 using surrogate virus neutralization test. Data represent mean ± s.e.m., *N* = 5 mice, one-way ANOVA followed by Dunnet’s multiple comparisons test. **D**) Body weight change follow-up in mice treated with siRNA NV-8. Data are presented as mean ± s.e.m. *N* until the endpoint (day 28): 10 mice per group. *N* after the endpoint: 5 mice per group.

**Figure S9.** **siPD-L1 NV-8 is safe *in vivo***. **A)** *In vivo* safety timeline, C57BL/6J mice. **B)** Body weight change, data represent mean ± s.e.m. *N* =10 mice. All group comparisons were non-significant (NS) by the two-way ANOVA test. **C**) Kaplan-Meier plot showing the probability of survival, *N* = 10 mice. **D-E**) Assessment of motor function. **D)** RotaRod test. The motor learning of C57BL/6 male mice was analyzed in a five-lane accelerating RotaRod. **E**) Open field test. The distance traveled by C57BL/6 male mice during a 15-min video recording was analyzed using EthoVision 13XT software. Data represent mean ± s.e.m., *N* = 10 mice. All group comparisons were NS by the One-way ANOVA test. One week after the last immunization, blood was collected, and nasal mucosa tissues were resected for additional analysis. **F-G**) Blood test, *N* = 5 mice. All comparisons between the groups were NS by the One-way ANOVA test. Data represent mean ± s.e.m. Whisker charts show minimum and maximum values. Blood chemistry panel (**F**) and Blood hematology panel (Complete blood count panel) (**G**). **H)** Representative images of H&E staining of mouse nasal mucosa. Images were captured by light microscopy, scale bar 100 μm.

Figure S10. Extracellular and intracellular staining of splenocytes. A representative gating strategy to evaluate cellular response on T cells.


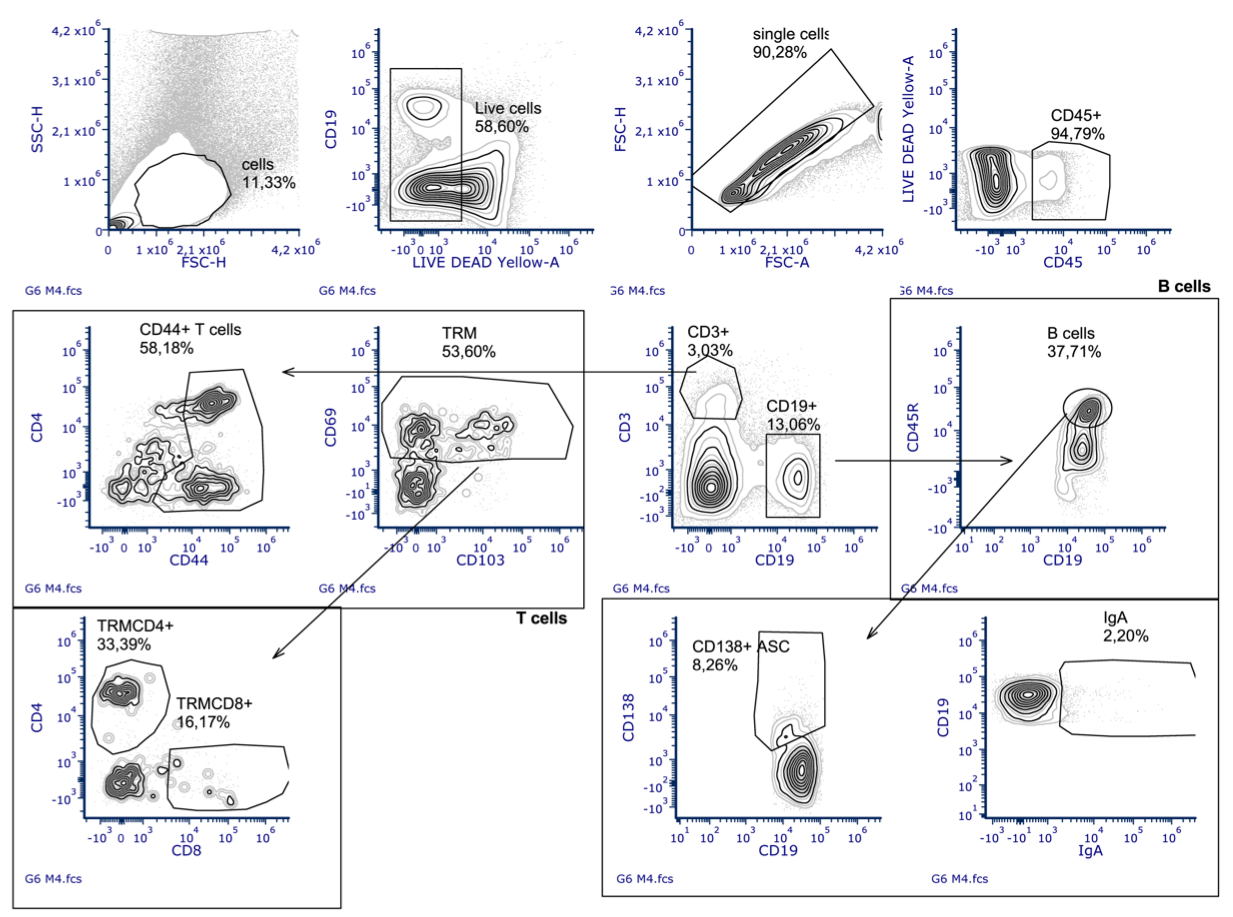


Figure S11. Extracellular staining of nasal mucosa cells. A representative gating strategy to evaluate mucosal immunity (tissue-resident T and B cells).
